# Adverse Drug Reactions of Olanzapine, Clozapine and Loxapine in Children and Youth: A Systematic Pharmacogenetic Review

**DOI:** 10.3390/ph15060749

**Published:** 2022-06-14

**Authors:** Diane Merino, Arnaud Fernandez, Alexandre O. Gérard, Nouha Ben Othman, Fanny Rocher, Florence Askenazy, Céline Verstuyft, Milou-Daniel Drici, Susanne Thümmler

**Affiliations:** 1 Department of Child and Adolescent Psychiatry, Children’s Hospitals of Nice CHU-Lenval, 06200 Nice, France; merino.d@chu-nice.fr (D.M.); fernandez.a@pediatrie-chulenval-nice.fr (A.F.); askenazy.f@pediatrie-chulenval-nice.fr (F.A.); 2 CoBTek Laboratory, Université Côte d’Azur, 06100 Nice, France; 3 Department of Pharmacology and Pharmacovigilance Center, University Hospital of Nice, 06000 Nice, France; gerard.a@chu-nice.fr (A.O.G.); ben-othman.n@chu-nice.fr (N.B.O.); rocher.f@chu-nice.fr (F.R.); pharmacovigilance@chu-nice.fr (M.-D.D.); 4 Service de Génétique Moléculaire, Pharmacogénétique et Hormonologie, Hôpital Bicêtre, Groupe Hospitalier Paris Saclay, AP–HP, 94270 Le Kremlin-Bicêtre, France; celine.verstuyft@aphp.fr; 5 CESP/UMR-S1178, Inserm, Université Paris-Sud, 92290 Paris, France

**Keywords:** cytochromes, *CYP1A2*, adverse drug reaction, antipsychotics, olanzapine, clozapine, loxapine, pharmacogenetics, children, youth

## Abstract

Children and youth treated with antipsychotic drugs (APs) are particularly vulnerable to adverse drug reactions (ADRs) and prone to poor treatment response. In particular, interindividual variations in drug exposure can result from differential metabolism of APs by cytochromes, subject to genetic polymorphism. *CYP1A2* is pivotal in the metabolism of the APs olanzapine, clozapine, and loxapine, whose safety profile warrants caution. We aimed to shed some light on the pharmacogenetic profiles possibly associated with these drugs’ ADRs and loss of efficacy in children and youth. We conducted a systematic review relying on four databases, following the Preferred Reporting Items for Systematic Reviews and Meta-Analyses (PRISMA) 2020 recommendations and checklist, with a quality assessment. Our research yielded 32 publications. The most frequent ADRs were weight gain and metabolic syndrome (18; 56.3%), followed by lack of therapeutic effect (8; 25%) and neurological ADRs (7; 21.8%). The overall mean quality score was 11.3/24 (±2.7). In 11 studies (34.3%), genotyping focused on the study of cytochromes. Findings regarding possible associations were sometimes conflicting. Nonetheless, cases of major clinical improvement were fostered by genotyping. Yet, *CYP1A2* remains poorly investigated. Further studies are required to improve the assessment of the risk–benefit balance of prescription for children and youth treated with olanzapine, clozapine, and/or loxapine.

## 1. Introduction

In child psychiatry, antipsychotic drugs (APs) are used to treat psychotic or mood disorders, as well as behavioral symptoms, despite limited evidence. Although APs are usually efficacious, the risk of adverse drug reactions (ADRs) associated with this class should be considered when initiating APs in this vulnerable population [1,2]. Treatment resistance is also a major concern [3]. Many intrinsic and extrinsic factors may influence the pharmacokinetics and pharmacodynamics of APs, such as sex, ancestry, puberty, dietary, and smoking habits [4,5,6,7], potentially leading to ADRs or lack of therapeutic effects.

Furthermore, the *cytochrome P450 (CYP)* proteins, a superfamily of liver enzymes, are instrumental to drug metabolism. At least 57 human *CYPs* have been described [8], even if most reactions are undertaken by *CYP2C9*, *CYP2C19*, *CYP2D6*, and *CYP3A4* [9]. Major interindividual differences in their expression arise from genetic polymorphisms, leading to various metabolizing phenotypes [10] that determine the *CYPs*’ level of activity. Furthermore, alterations in their activity by extrinsic inducers or inhibitors, can imbalance a previously well-tolerated treatment; conversely, it can potentiate a given medication [11]. As *CYP* metabolize most APs [12], some studies addressed the potential consequences of *CYP2D6* polymorphisms in children and youth treated with antipsychotics [13]. While *CYP1A2* represents approximately 15% of hepatic *CYP* content [14], it is nonetheless pivotal in the metabolism of the two atypical APs, olanzapine [15] and clozapine [16], as well as loxapine [17] (whose properties are closely related to those of atypical APs [18,19]).

Olanzapine, clozapine, and loxapine share a common tricyclic structure and belong to the thienobenzodiazepine, dibenzodiazepine, and dibenzoxazepine families, respectively [20]. Olanzapine [21] and clozapine [22] are currently used as second- to third-line therapy, while loxapine may allow symptomatic relief of acute agitation [23,24]. In child psychiatry, the Food and Drug Administration (FDA) has granted marketing authorization for olanzapine in acute mixed or manic episodes of bipolar I disorder and treatment of schizophrenia for adolescents aged from 13 to 17 years old [25]. Similarly, the FDA authorized use of olanzapine in cases of depressed bipolar I disorder, in combination with fluoxetine, in children and adolescents aged between 10 and 17 years old [25]. By contrast, the European Medicines Agency (EMA) did not recommend olanzapine for use in children and adolescents below 18 years of age, mainly because of a lack of data on safety and efficacy. Furthermore, the EMA highlighted a greater magnitude of weight gain, lipid, and prolactin alterations in short-term studies of adolescent patients, in comparison with studies of adult patients [26]. Regarding clozapine, its therapeutic indications are mainly represented by treatment-resistant schizophrenia and recurrent suicidal behaviors in schizophrenic disorders [27], without prejudice to the age, reflecting the lack of guidelines for use of clozapine in pediatric population [28]. The EMA stated that safety and efficacy of clozapine in children under the age of 16 have not been established yet, and therefore that it should not be used in this group until further data become available [29]. Likewise, regarding loxapine, both FDA and EMA mentioned that safety and effectiveness in pediatric patients have not been established [30,31]. However, in France, the National Drug Agency (Agence Nationale de Sécurité du Médicament et des produits de santé (ANSM)) granted authorization for loxapine in the treatment of acute and chronic psychotic disorders as from the age of 15 years [32].

Atypical APs tend to induce less extrapyramidal effects (compared to typical antipsychotics) [33] and may therefore be the preferred option when treating children and youth, despite these grey areas. However, their profile comes at the price of other prominent ADRs, such as metabolic changes (weight gain, hyperglycemia, and dyslipidemia) [34]. As they begin in childhood, they are likely to persist over lifetime. Off-label use being frequent in this population [35], children are also exposed to a plethora of ADRs, such as neuroleptic malignant syndrome, seizures, agranulocytosis, or hyperprolactinemia. The safety profile of olanzapine [36] and clozapine [1] shows major issues of concern, and the tolerability of loxapine scarcely has been investigated [37], especially in children and youth.

Increased knowledge of the intrinsic determinants of each patient’s exposure to APs could pave the way to tailored therapy. Pharmacogenetics has been defined as the study of how genetic differences influence the variability in patient’s responses to drugs [38]. On a large scale, genome-wide association studies (GWAS) allow to genotype all known single-nucleotide polymorphisms (SNPs) in the human genome. When a smaller set of SNPs are likely to affect treatment response, candidate gene studies can be conducted to detect a potential association [39]. Further, whole-genome sequencing approaches (WGS) may allow to identify rare gene variants, and therefore raises interesting prospects in psychiatric disorders [40,41]. The *in vivo* assessment of a cytochrome’s phenotype relies on the administration of a selective enzyme substrate. These approaches brought us closer to personalized medicine, whereby the understanding of each patient’s genetic profile may predict the occurrence of ADRs or lack of effect. This may be especially useful in specific populations [42], often excluded of clinical trials and of the classical field of evidence-based medicine.

Therefore, we aimed to review the pharmacogenetic variants underlying olanzapine, clozapine, and loxapine ADRs and/or efficacy in children and youth having undergone genotyping. Then, we assessed the most frequently investigated ADRs and genetic polymorphisms in this population. Finally, we assessed the specific effect of *CYP1A2* variants in the occurrence of ADRs and/or lack of therapeutic effect.

## 2. Materials and Methods

### 2.1. Research

The PROSPERO International prospective register of systematic reviews was checked for similar systematic reviews. Due to our issue of concern never having been addressed, we have submitted the research protocol to the INPLASY International platform of registered systematic review and meta-analysis protocols (INPLASY202250025).

We have, therefore, conducted this systematic review following the Preferred Reporting Items for Systematic Reviews and Meta-Analyses (PRISMA) 2020 recommendations and checklist [43]. We further followed special methodological considerations regarding pediatric systematic reviews [44]. The following query was used: ((((adolescent* OR youth OR child* OR pedia* OR paedia*) AND (clozapine OR olanzapine OR loxapine) AND (pharmacogen* OR allele OR genotype* OR cytochrome* OR CYP1* OR CYP2* OR CYP3* OR CYP4*) AND (adverse drug reaction* OR adverse event* OR adverse reaction* OR side effect* OR secondary effect* OR after effect* OR tolerability OR safety)))). Two authors (D.M. and A.O.G.) separately conducted the research in PubMed, EMBASE, PsycINFO, and PsycArticles. Our query retrieved publications registered in the four selected databases up to 21 March 2022.

**Figure 1:**
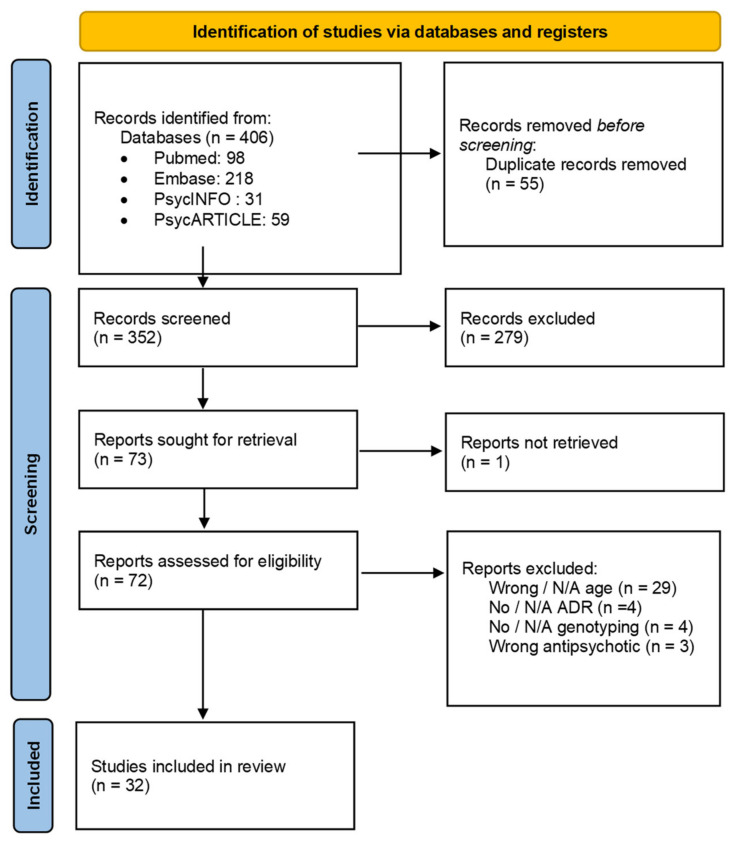
PRISMA 2020 flow diagram for identification of studies. N/A: Not applicable. From: Page MJ, McKenzie JE, Bossuyt PM, Boutron I, Hoffmann TC, Mulrow CD, et al. The PRISMA 2020 statement: an updated guideline for reporting systematic reviews. BMJ 2021; 372: n71. doi:10.1136/bmj.n71.

Relying on four electronic bibliographic databases, our extraction retrieved for each publication the source database, publication year, language, full list of authors’ names, article title, DOI (Digital Object Information), journal title, abstract, and Medical Subject Headings (MeSH) terms associated. Two authors independently performed the preliminary two steps of proper article screening, with the results shown in the PRISMA flowchart (Figure 1).

Before screening, duplicates were removed. First, the eligibility of the titles and abstracts of the articles identified by the initial query were checked. Next, full-text copies of the articles whose titles and abstracts met the inclusion criteria were retrieved. Then, to ensure compliance with the inclusion criteria, the yielded full-text articles were assessed for eligibility.

When the two reviewing authors could not obtain a consensus regarding an article, the disagreement was resolved through discussion. Lastly, data extraction was performed for all publications that met the inclusion criteria, including the study site(s), study type, characteristics of the subjects (age, sample size, sex distribution, ancestry, diagnosis), antipsychotic(s) of interest and its (their) dosing, other drugs administered, outcome(s) measured, gene variants assessed, their potential association(s) with the ADR(s), the pathophysiology involved, and the pharmacogenetic approach. For quality assessment needs, we also extracted data addressing the reasons for choosing the genes/SNPs to genotype (summaries of previous findings, reasons given for choosing the genes and SNPs genotyped, the adjustment methods for multiple testing, and the *p*-values provided for the associations), the sample size (details on calculation of sample size and on a priori power to detect effect sizes of varying degrees), the reliability of genotypes (description of the genotyping procedure, of the primers and of any quality control methods, previously reported genotype frequencies, blind of genotyping personnel to outcome status), missing genotype data (the extent and reasons for missing data, any checks for missingness at random performed, any imputation of missing genotype data, number of patients contributing to each analysis and consistence with sample size), population stratification (tests undertaken for cryptic population stratification and adjustment for in the analyses), Hardy–Weinberg Equilibrium testing (was it performed, and were deviating (or not) SNPs highlighted and excluded from further analysis where appropriate), and choice and definition of outcomes (clear definition of all outcomes investigated, justification, results shown).

### 2.2. Selection Criteria

Data extraction relied on the following inclusion criteria:

1. Studies including at least one child and/or adolescent and/or youth, therefore aged under 25, following the United Nations definition [45].
2. Receiving at least one atypical antipsychotic that is metabolized by *CYP1A2* (clozapine, olanzapine, loxapine).
3. Having experienced an adverse drug reaction/a lack of therapeutic effect linked to at least one of these treatments.
4. Having undergone pharmacogenomic analysis/genotyping, the results of which are mentioned.
5. Record issued from an English-language and peer-reviewed journal, for which full-text was available

We therefore excluded books (and chapters), commentaries, but also any published material that did not meet the original research criteria (e.g., systematic reviews, meta-analyses) [46]. However, considering the foreseeable paucity of evidence informing the review, we decided to include conference abstracts and editorial pieces [47].

To serve the same purpose, we have chosen to include studies including ‘mixed’ (both adult and pediatric) populations [44], with due regard to the age criterion: ‘Studies including at least one child and/or adolescent, therefore aged under 25′.

Then, identical or overlapping patient cohorts were detected by the analysis of study site(s) and characteristics of the subjects, among others. The objectives and genetic variants investigated tended to differ across the reports, based on overlapping or identical cohorts, so we have chosen to include publications presenting redundant cohorts [39].

When the ancestry of patients (whose consideration is pivotal in genetics concerns) was not provided in a study, we hypothesized that it could be consistent with the study site, and reported it as such.

Studies were classified according to their methodology: case reports or case series, cohort studies [48], and case–control (or cross-sectional) studies [49]. We distinguished ‘pediatric’ studies, exclusively relying on pediatric samples, and ‘mixed-population’ studies, to present their respective characteristics (Table 1 and Table 2) and quality assessments (Tables S1 and S2). Then, the whole studies were grouped according to the main classes of ADRs investigated (Table 3, Table 4 and Table 5).

### 2.3. Quality Assessment

The quality of the included pharmacogenetic studies was independently assessed by D.M. and A.O.G, relying on a tool adapted from Maruf et al. [13] and the checklist developed by Jorgensen and Williamson [50]. As stated above, we considered each article (irrespective of the potential redundancy of its (their) cohort(s)) for quality assessment. Indeed, methods may vary from an article to another, relying on identical or overlapping patient cohorts. Any case of discrepancy between their assessments was resolved through discussion.

The used tool addressed different issues of methodological quality:

1. Choosing the genes/SNPs to genotype (4 binary questions).
2. Sample size (3 questions: 2 binary and 1 open).
3. Study design (1 open question).
4. Reliability of genotypes (5 binary questions).
5. Missing genotype data (6 binary questions).
6. Population stratification (2 binary questions).
7. Hardy–Weinberg Equilibrium (2 binary questions).
8. Choice and definition of outcomes (3 binary questions).

The purpose of open questions (sample size; study design) was to allow a quality visual check as a complement to the global score of each publication.

For each binary question, we answered:

- ‘Yes’ if the study provided an adequate response.
- ‘No’ if the response was not mentioned in the manuscript nor a method publication referenced by the authors.
- ‘N/A’ (not applicable) if the response to the main (first) question of the issue of concern addressed is ‘No’.

Consequently, each study received a quality score between 0 and 24, based on the summation of the ‘Yes’ answers. According to this approach, the higher the score, the higher the quality of a given study.

## 3. Results

### 3.1. Study Selection

Selection and progressive elimination of the identified articles are summarized in the Preferred Reporting Items for Systematic Reviews and Meta-Analyses (PRISMA) flowchart provided in Figure 1. Our database query retrieved 406 records. Before screening, we removed 55 duplicates (see Methods). Then, 352 records were screened on the basis of their title and abstract. Among them, 72 publications were assessed for eligibility via the analysis of their full-text version. Finally, 32 records met the inclusion criteria of this systematic review.

### 3.2. Characteristics of Studies

#### 3.2.1. General Characteristics

The most represented study type was cohort studies (20 reports; 62.5%). Sample sizes ranged from single cases (case reports) to 1445 patients (case–control study). Among articles for which the ancestry was provided, 90.9% involved Caucasian/European/White populations. It was not reported in 10 records (31.3%). Diagnosis of the included patients was provided in 32 records (96.9%), mainly represented by psychotic disorders (29 reports; 93.5%). In 11 studies (34.3%), genetic assessment relied on studying cytochromes. Olanzapine was the most commonly used AP (24 reports; 75.0%). The most frequent ADR was weight gain and metabolic syndrome (MetS), investigated in more than half of the studies (18 reports; 56.3%). Lack of therapeutic effect accounted for 8 reports (25.0%) and neurological ADRs for 7 reports (21.8%). Comparing study sites and characteristics of the populations, we noticed several overlaps between the included articles. Indeed, Nussbaum et al. in both studies ([51,52]), as well as Le Hellard et al. [53] and Jassim et al. [54] relied on identical cohorts, respectively. To a lesser extent, Le Hellard et al. included the Theisen et al. [55] cohort; the Gagliano et al. [56] cohort overlapped with the Tiwari et al. [57] cohort; and the Quteineh et al. [58] and Saigi et al. [59] cohorts were both overlapping the Choong et al. [60] cohort.

The mean quality assessment score (see Methods) of the 32 included studies was 11.3/24 (±2.7). The scores ranged from 6 (a case series) to 18 (a cohort study). In all studies, a literature review was undertaken, whose findings were summarized, as well as the reasons for choosing the genes and SNPs genotyped. The method of adjustment for multiple testing was described in 13 records (40.6%). Precise *p*-values were provided for all associations in 25 records (78.1%). Regarding sample size, details on its calculation were given in one (3.1%) study (a cohort study). Details were given regarding the a priori power to detect effect sizes of varying degrees in 5 publications (15.6%). Almost all records described the genotyping procedure (31; 96.9%). Primers and quality control methods were described in 8 (25.0%) and 6 (18.8%) studies, respectively. Previously reported genotype frequencies were quoted in 9 publications (28.1%). Genotyping personnel was blinded to outcome status in one study (a cohort study) (3.1%). The extent of missing data was summarized in 9 studies (28.1%), among which 6 gave the reasons for missing data (66.7%). No study reported checks for missingness at random, nor imputed missing genotype data. All studies quoted the number of patients contributing to each analysis (32; 100%), which agreed to samples sizes in 24 studies (75.0%). No study presented tests for cryptic population stratification. Hardy–Weinberg Equilibrium (HWE) was tested in 18 reports (56.3%). Among them, the presence (or the absence) of deviating SNPs was highlighted and excluded from further analysis in 17 studies (94.4%). Finally, all studies provided definitions, justifications for their choices, and results for all outcomes investigated (32; 100%).

#### 3.2.2. Pediatric Studies

Cohort studies accounted for 41.6% of pediatric studies (*n* = 5), followed by case reports and case series (4 studies; 33.3%). Sample sizes ranged from single cases (2 case reports) to 279 patients (a cohort study). The population was aged 3 to 20 years old. Ancestry was not reported in most publications (7 studies; 58.3%). All studies in which ethnicity was reported included Caucasian/European/White populations and African/Black populations (5; 100%). Patients’ diagnosis was mentioned in 11 studies (91.6%); psychotic disorders in 8 of them (72.7%) and mood disorders in 5 of them (45.5%). Cytochromes were genotyped in a great majority of reports (9; 75.0%). Olanzapine was mentioned in nearly all the publications (11; 91.6%). Among the studied ADRs, 5 studies were related to inadequate efficacy (41.7%), 4 (33.3%) to weight gain or MetS, and 3 (25.0%) to neurological symptoms. Detailed characteristics of the included pediatric studies are provided in Table 1.

For pediatric studies, the average quality assessment score was 9.1/24 (±1.7), ranging from 6 (a case series) to 13 (a cohort study). The adjustment for multiple testing was described in one-fourth of the studies (3; 25.0%), and precise *p*-values were provided for all associations in one-half of the studies (6; 50.0%). No pediatric study provided details on the calculation of the sample size nor on the a priori power to detect effect sizes of varying degrees. The genotyping procedure was described in nearly all the publications (11; 92.0%). However, no study described the primers nor the quality control methods used. Previously reported genotype frequencies were quoted in 4 studies (33.3%). No study reported blinding of the genotyping personnel to outcome status. One study (1; 8.3%) summarized the extent of missing data (a cohort study), but justifications were not provided. The number of patients contributing to analyses agreed to the sample size in 10 studies (83.3%). HWE was tested in one study (a cohort study), where the absence of deviation was highlighted (1; 8.3%). The comprehensive quality assessment for pediatric studies is displayed in Table S1.

**Table 1:** Characteristics of the studies (pediatric population).

Study	Design	N	Age (Years)	Male (%)	Ancestry	Diagnosis	Antipsychotic	Gene Variant	ADR	Quality
Baumann et al. (2006)	Case Report	1	14	0	Swiss?	OCD	Olanzapine	CYP2D6 XN; *4; CYP3A5 *3; CYP2B6 *6; CYP2C9 *1; CYP2C19 *1	Generalized tonic–clonic seizure	8
Prows et al. (2009)	Cohort study	279 (18 OLZ)	3 to 18; mean (12.7 ± 3.2)	50.9%	White 72.4%; Black 22.6%; Other 5.0%	Mood disorders; Disruptive behavior; Anxiety, ICD; Psychotic disorders; PDD; ED; Adjustment disorders; Other	Olanzapine	CYP2D6 *1, *3, *4, *5, Dup; CYP2C19 *1, *2	Sleep disturbances; gastro-intestinal symptoms; headache, difficulty concentrating; mood change; dizziness; extrapyramidal symptoms; aggressive behavior; rash; shortness of breath; lack of therapeutic effect	9
Devlin et al. (2012)	Case–control study	105 (4 OLZ)	mean (12.58 ± 3.14)	66.7%	European 74%; Asian 8.7%; Aboriginal 2.9%; South Asian 2.9%; African/Caribbean 10.7%; Hispanic 4.8%	Non provided	Olanzapine	*MTHFR (rs1801133) C677T C;T*	Metabolic syndrome	9
Nussbaum et al. (2014)	Cohort study	81	9 to 20; median (15.74)	46%	Romanian?	Schizophrenia; BD	Olanzapine	CYP2D6 *4	Weight gain	9
Nussbaum et al. (2014)	Cohort study	81	9 to 20; median (15.74)	46%	Romanian?	Schizophrenia; BD	Olanzapine	CYP2D6 *4	Lack of therapeutic effect	8
Butwicka et al. (2014)	Case Report	1	16	100%	Polish?	Schizophreniform disorder	Olanzapine	CYP2D6 *4	Neuroleptic Malignant Syndrome	8
Cote et al. (2015)	Case–control study	134 (5 OLZ)	mean (12.5 ± 3.1)	68.7%	European 73.9%; African 7.5%; Asian 9.0%; Hispanic 5.2%; South Asian 2.2%; First Nations 2.2%	Anxiety, Depression, ADHD, Mood disorder, Psychotic disorder, Adjustment disorder, PDD, Other	Olanzapine	*COMT Val158Met (rs4680) Met; Val*	Cardiometabolic risk factors	10
Ocete-Hita et al. (2017)	Case–control study	92: 30 cases (1 OLZ); 62 controls	0 to 15; mean (8.3 ± 3)	36.7%	White 90%; Black 3.3%; Other 6.6%	ADHD	Olanzapine	Class I HLA-A, B, C* loci, *class II HLA-DRB1, DQB1, DQA1, DP* loci, *KIR: 14 KIR* genes and 2 *pseudo-KIR* genes; *TNFα (rs1800629); TGFβ1 (-10T/A; 25C/G); IL-10 ((rs1800896); -819T/C; -(rs1800872)); IL-6 (rs1800795); IFNγ (rs2430561)*	DILI: Idiosyncratic Drug-Induced Liver Injury	10
Thümmler et al. (2018)	Case series	9 (3 OLZ, CLZ, LOX)	11 to 16; mean (14.1 ± 1.8) (13 to 16 OLZ, CLZ, LOX)	55.5% (33% OLZ, CLZ, LOX)	French?	COS, ASD, ODD (OLZ, CLZ, LOX); COS, PTSD, behavioral disorder, ASD, ODD, ID	Olanzapine; Clozapine; Loxapine	CYP2D6 *3, *4, *5, *6, *41, Dup	EPS, weight gain, hepatic cytolysis, akathisia, dystonia, galactorrhea, binge eating, weight gain, constipation, lack of therapeutic effect	9
Grădinaru et al. (2019)	Cohort study	81	9 to 20; median (15.74)	54%	Romanian?	Schizophrenia; BD	Olanzapine	CYP2D6 *3, *4, *5, *41	Hyperprolactinemia	10
Ivashchenko et al. (2020)	Cohort study	53 (6 CLZ) (5 OLZ)	mean (15.08 ± 1.70)	52.8%	Russian?	BPD; schizophrenia; schizoaffective disorder; schizotypal disorder; MDD; delusional disorders	Clozapine; Olanzapine	CYP2D6 *4, *9, *10; CYP3A4 *22, CYP3A5 *3; *ABCB1 (rs1128503, rs2032582, rs1045642)*; *DRD2 (rs1800497); DRD4 (rs1800955); HTR2A (rs6313)*	Lack of therapeutic effect; decreased/increased salivation, increased/reduced duration of sleep, tremor, constipation, subjective akathisia; polyuria/polydipsia; increased dream activity	13
Berel et al. (2021)	Case series	4	9; 10; 11; 14;	75%	2 Caucasian, 1 Caucasian/Indian, 1 African	Tourette syndrome and ID; behavioral disorders and neurodevelopmental delay; EOS; ASD with catatonia	Clozapine	CYP1A2 *1F, *1; CYP2D6 *1, *4, *10, *41; CYP2C19 *1, *2; CYP3A5 *1, *3; CYP3A4 *1; CYP2C9 *1, *3	Lack of therapeutic effect (low concentrations)	6

OLZ: Olanzapine; CLZ: Clozapine; LOX: Loxapine; OCD: Obsessive Compulsive Disorder; ICD: Impulse Control Disorder; PDD: Pervasive Development Disorder; ED: Eating Disorder; ADHD: Attention Deficit Hyperactivity Disorder; COS: Childhood Onset Schizophrenia; ASD: Autism Spectrum disorder; ODD: Oppositional Defiant Disorder; ID: Intellectual Disability; PTSD: Post-Traumatic Stress Disorder; BDP: Brief Psychotic Disorder; MDD: Major Depressive Disorder; EOS: Early Onset Schizophrenia; EPS: Extrapyramidal Syndrome ?: when the ancestry of the patients was not provided in a study, we hypothesized that it could be consistent with the study site, and reported it as such.

#### 3.2.3. Mixed Population Studies

Among mixed-population studies, cohort studies were prevailing (15; 75.0%). The sample sizes ranged from 21 to 1445 (both case–control studies). Age ranged from 10 to 75 years old. Ancestry was available in 17 reports (85.0%), among which Caucasian/European/White populations accounted for 88.2% (15 reports). All studies included patients suffering from schizophrenia-spectrum disorders (20 reports; 100%). Serotonin receptors or transporters, genes coding for proteins involved in energy and lipid homeostasis, and *COMT Val158Met (rs4680)* polymorphism were assessed in 3 studies each (15.0%). Regarding antipsychotics of interest, 15 studies involved clozapine (75.0%), and 13 studies involved olanzapine (65.0%). Weight gain and MetS were studied in 14 studies (70.0%), followed by lack of therapeutic effect (3; 15.0%) and extrapyramidal syndrome (EPS) (2; 10.0%). Detailed characteristics of the mixed population studies are provided in Table 2.

For mixed population studies, the mean quality assessment score was 12.6/24 (± 2.4), lying between 8 (a case–control study) and 18 (a cohort study). The method used to adjust for multiple testing was described in one-half of the studies (10; 50.0%). Precise *p*-values were provided for all associations in almost all studies (19; 95.0%).The calculation of sample size was detailed in one study (1; 5.0%) and the a priori power to detect effect sizes of varying degrees was detailed in 5 studies (5; 20.0%). All studies described the genotyping procedure (20; 100%). Primers were described in 8 studies (40.0%), and quality control methods in 6 studies (30.0%). Previously reported genotype frequencies were quoted in one-fourth of the studies (5; 25.0%). Genotyping personnel was blinded to outcome status in one study (a cohort study) (5.0%). The extent of missing data was summarized in 8 reports (40.0%), among which 6 justified it (75.0%). The number of patients contributing to the analyses agreed to sample size in 14 studies (70.0%). HWE was tested in 17 reports (85.0%), among which almost all (16; 94.1%) underlined the presence (or absence) of deviating SNPs and excluded them from further analysis when appropriate. The comprehensive quality assessment for mixed population studies is displayed in Table S2.

**Table 2:** Characteristics of the studies (mixed population).

Study	Design	N	Age (Years)	Male (%)	Ancestry	Diagnosis	Antipsychotic	Gene Variant	ADR	Quality
Vandel et al. (1999)	Case–control study	65: 22 cases (1 OLZ); 43 controls	16 to 75; mean (41.9 ± 1.9)	35%	French?	MDD, dysthymia, OCD, schizophrenia	Olanzapine	CYP2D6 *1A, *2, *2B, *3, *4A, *4D *5, *6B, *9, *10B	EPS: akathisia, dystonia, parkinsonism, dyskinesia	8
Hong et al. (2002)	Cohort study	88	18 to 66; mean (37.1 ± 8.2)	66%	Han Chinese	schizophrenic disorders	Clozapine	*H1 receptor (rs2067467) Glu, Asp*	Weight gain	11
Mosyagin et al. (2004)	Case–control study	159: 81 cases (49 CLZ), (2 OLZ); 78 controls	Female: 22 to 85; mean (48); Male: 18 to 77; mean (47)	36%	German Whites	schizophrenia paranoid type	Clozapine, Olanzapine	*MPO (rs2333227) G,A; CYBA (rs4673) C,T; (rs1049255) A,G*	Agranulocytosis	13
Theisen et al. (2004)	Cohort study	97	14 to 45; mean (22.1 ± 7.7)	59%	German	schizophrenia spectrum disorders	Clozapine	*5-HT2CR (rs3813929)-759C/T C,T*	Weight gain	11
Kohlrausch et al. (2008)	Cohort study	121: (55 NR), (27 NOGS)	16 to 64: mean (34.02 ± 8.79) total; mean (34.13 ± 9.84) NR; mean (34.37 ± 9.41) NOGS	total 83.5%; NR 81.8%; NOGS 70.4%	European	schizophrenia	Clozapine	*GNB3 (rs5443) 825C > T*	Lack of therapeutic effect, NOGS: new onset generalized seizures	12
Godlewska et al. (2009)	Cohort study	107	mean (29.3 ± 10.0)	49%	Caucasian, Polish	schizophrenia (mostly paranoid)	Olanzapine	*5-HT2CR (rs3813929) 759C/T C,T;* *5-HT2CR (* *rs518147) 697G/C G,C*	Weight gain	13
Le Hellard et al. (2009)	Cohort study	160	10 to 64; mean (21.9 ± 8.9)	61%	German	schizophrenia spectrum disorders	Clozapine	*44 SNPs: 3 SNPs in INSIG1; 21 SNPs in INSIG2; 3 SNPs in SCAP; 4 SNPs in SREBF1; 13 SNPs in SREBF2*	Weight gain	14
Tiwari et al. (2010)	Cohort study	183	18 to 60; mean (36.12 ± 10.17)	67.8%	European-American 63.9%; African-American 30.1%; Others 6.0%	schizophrenia or schizoaffective disorders	Clozapine, Olanzapine	*20 SNPs in CNR1*	Weight gain	17
Lencz et al. (2010)	Cohort study	58	16 to 38; mean (23.5 ± 4.9)	76.8%	African-American 40%; Caucasian (European) 28%; Hispanic 19%; Asian 5%; Other 8%	schizophrenia, schizoaffective or schizophreniform disorder	Olanzapine	*DRD2 (rs1799732)* *141C Ins; Del*	Weight gain	12
Kohlrausch et al. (2010)	Cohort study	116 (52 NR)	16 to 64; mean (33.82 ± 8.51)/R: mean (33.89 ± 8.04)/NR: mean (33.73 ± 9.14)	85.3%/R 85.9%/NR 84.6%	European	schizophrenia	Clozapine	*5-HTT HTTLPR (rs25531) LA, LG, S; VNTR Stin2 9, 10, 12 repeats*	Lack of therapeutic effect	11
Jassim et al. (2011)	Cohort study	160	10 to 64; mean (21.9 ± 8.9)	61%	Central European	schizophrenia spectrum disorders	Clozapine	*96 SNPs: 13 for ADIPOQ; 10 for FABP3; 7 for PRKAA1; 14 for PRKAA2; 3 for PRKAB1; 4 for PRKAG1; 40 for PRKAG2; 4 for PRKAG3; 1 for FTO*	Weight gain	12
Choong et al. (2013)	Cohort study	444; S1: 152; S2: 174; S3: 118	S1: 19 to 64, median (42); S2: 12 to 69, median (35); S3: 19 to 69, median (42)	S1: 52%; S2: 49%; S3: 67%	Swiss?	Psychotic disorders, mood disorders, others	Clozapine, Olanzapine	*3 CRTC1 SNPs: rs10402536 G > A; rs8104411 C > T; rs3746266 A >G*	Weight gain	13
Gagliano et al. (2014)	Cohort study	99	18 to 65 median (34)	44%	Caucasian	schizophrenia or schizoaffective disorders	Clozapine, Olanzapine	*16 PRKAR2B SNPs*	Weight gain	18
Dong et al. (2015)	Cohort study	536: D: 328; R: 208	D: 18 to 45 mean (29.1 ± 7.6); R: 18 to 60 mean (21.3 ± 8.2)	D: 48.7%; R 57.2%	Chinese Han	schizophrenia	Olanzapine	*4 A2BP1 SNPs: rs10500331, rs4786847, rs8048076, rs1478697, rs10500331*	Weight gain	14
Pouget et al. (2015)	Case–control study	1445: 670 cases; 775 controls	18 to 60; (38.54 ± 10.4)	71%	European	schizophrenia of schizoaffective disorders	Clozapine, Olanzapine	*TSPO 8 SNPs: rs739092, rs5759197, rs138911, rs113515, rs6971, rs6973, rs80411, rs138926*	Weight gain; lack of therapeutic effect	16
Quteineh et al. (2015)	Cohort study	834: 478 + 168 + 188	main: 12 to 97 median 50; S1 19.5 to 64, median (42.2); S2: 19 to 69, median (42.3)	main: 43.7%; S1 52.9%; S2 62.2%	White	Psychotic disorders, mood disorders, schizoaffective disorders, others	Clozapine, Olanzapine	*HSD11B1 7 variants: rs12565406 G > T, rs10863782 G > A, rs846910 G > A, rs375319 G > A, rs12086634 T > G, rs4844488 A > G, rs84690 C > T*	MetS	11
Saigi et al. (2016)	Cohort study	750: S1: 425; S2:148; S3: 177	combined 13 to 97 median 45; S1 13 to 97 median 51; S2 19 to 64 median 42; S3 18 to 69 median 42	combined 50%; s1 43% s2 55% s3 62%	White	psychotic disorders, schizoaffective disorders, BD, depression, other	Clozapine, Olanzapine	52 SNPs previously associated with BMI/21 associated with type 2 diabetes/9 associated with psychiatric disorders	Weight gain	14
Nelson et al. (2018)	Case–control study	71: cases 32 (1 OLZ); controls 39	15 to 55 Met FEP mean 25.15 ± 7.20, Val FEP mean 22.92 ± 7.08	FEP Met 75%; FEP Val 58%	Caucasian, African American, Other	schizophrenia spectrum, BD with psychosis, MDD with psychosis, psychotic disorder NOS	Olanzapine	*COMT Val158Met (rs4680) Met; Val*	alteration of cognitive flexibility	11
Menus et al. (2020)	Cohort study	96	18 to 74, median (39)	40%	Hungarian?	schizophrenia	Clozapine	CYP1A2 *1C, *1F, *1; CYP3A5 *1, *3; CYP3A4 *1, *1B, *22	MetS, altered concentration, hypersalivation, blurred vision, constipation, fatigue	11
Nicotera et al. (2021)	Case–control study	21: 4 cases; 17 controls	16 to 46	62%	Caucasian	ID, psychotic disorder, schizophrenia spectrum, gait disorder, specific learning disorder, schizotypal personality disorder	Clozapine, Olanzapine	*COMT Val158Met (rs4680) Met; Val COMT L136L (rs4818) G,C*	Dystonia	11

OLZ: Olanzapine; CLZ: Clozapine; NR: Non responders; FEP: First episode psychosis; OCD: Obsessive Compulsive Disorder; ID: Intellectual Disability; MDD: Major Depressive Disorder; BD: Bipolar Disorders; SNP: Single-Nucleotide Polymorphism; EPS: extrapyramidal syndrome; MetS: Metabolic Syndrome. Ancestry: ‘?’ when the ancestry of the patients was not provided in a study, we then hypothesized that it could be consistent with the study site, and reported it as such.

### 3.3. Main Adverse Drug Reactions

#### 3.3.1. Weight Gain and Metabolic Syndrome 

While 14 studies (43.8%) investigated solely weight gain, 4 studies (12.5%) addressed the potential correlations of MetS with genetics, as shown in Table 3. Among studies specifically assessing antipsychotic-induced weight gain (AIWG), 2 were pediatric studies (14.3%) and 12 were mixed-population studies (85.7%). Both pediatric and mixed studies accounted for half (2; 50.0%) of the reports addressing MetS.

In 2014, Nussbaum et al. [51] found that CYP2D6 wt/*4 (intermediate metabolizer–IM) children had a significant increase in weight gain when compared to the patients without *4 allele, after six months of administration of atypical APs (*p* < 0.001). Likewise, Thümmler et al. [3] reported the case of a CYP2D6 *4/*41 (poor metabolizer–PM) 14-year-old female who showed weight gain and binge-eating behaviors when treated with clozapine and loxapine. According to the findings of Menus et al. [61], a moderate/high risk of obesity in patients treated with clozapine was significantly more frequent in *low CYP3A4 expressers* (13.6% of *CYP3A4 low expressers*, 1.5% of *CYP3A4 normal/high expressers*, OR = 13.5 (95% CI 1.2–147.9), n = 87, *p* = 0.045). However, there was no association between *CYP1A2* or *CYP3A4* expression and blood glucose or lipid levels (*p* > 0.1). By contrast, in *low CYP3A4 expressers*, a significant correlation was found between the clozapine serum concentration and blood glucose level (r = 0.52, n = 20, *p* = 0.02).

Few studies investigated the potential link between lipid homeostasis and polymorphisms of genes involved in energy. Indeed, Le Hellard et al. [53] found a strong association (*p* = 0.0003–0.00007) between three genetic polymorphisms localized within or near the *INSIG2* gene *(rs17587100, rs10490624*, and *rs17047764)* and AIWG in patients treated with clozapine. Choong et al. [60] found that carriers of the *CRTC1 (rs3746266) G allele* had a lower BMI than noncarriers *(AA genotype)* (*p* = 0.001, *p* = 0.05, and *p* = 0.0003, respectively, in the three samples). When excluding patients taking other weight gain-inducing drugs, *G allele* carriers (*n* = 98) had a 1.81 kg/m^2^ lower BMI than noncarriers (*n* = 226; *p* < 0.0001). This association was more marked in women aged under 45 years, with a 3.87 kg/m^2^ lower BMI in *G allele* carriers (*n* = 25) compared with noncarriers (*n* = 48; *p* < 0.0001). In patients treated with clozapine, Jassim et al. [54] found a marked association between AIWG and 6 genetic polymorphisms in *ADIPOQ*, among which only 2 showed both allelic and genotypic association. Body Mass Index (BMI) changes were, to a lesser extent, associated with one marker in *PRKAA1 (rs10074991)*, by an allelic (*p* = 0.011) and genotypic (*p* = 0.004) association, as well as three markers in *PRKAA2 (rs4912411*, *p* = 0.044; *rs7519509*, *p* = 0.043; *rs10489617*, *p* = 0.036). In *PRKAG2*, one marker (*rs17714947*, *p* = 0.020) displayed allelic association with AIWG, while another marker (*rs7800069*, *p* = 0.0008) showed genotypic association. By contrast, Gagliano et al. [56] analyzed 16 tag SNPs across the *PRKAR2B* gene in a sample of patients treated with clozapine or olanzapine. Patients displaying the minor allele of the polymorphism *PRKAR2B (rs9656135)* had a mean weight increase of 4.1%, whereas patients without this allele had an increase of 3.4%, but this association did not remain significant after correcting for multiple testing. Quteineh et al. [58] found that only male carriers of the *HSD11β1 (rs846906) T allele* had significantly higher waist circumference and triglycerides (TG), and lower high-density lipoprotein cholesterol (HDL) (*p*_corrected_ = 0.028). This allele was also associated with a higher risk of antipsychotic-induced MetS at 3 months of follow-up (OR = 3.31 (95% CI 1.53–7.17), *p*_corrected_ = 0.014). When studying patients treated with APs, the impact of 52 SNPs previously associated with BMI changes, Saigi et al. [59] found that *CADM2 (rs13078807)* showed a nominal association with BMI over time (*p* = 0.01), with a 1.04 increase in BMI per additional risk allele after 12 months of treatment. The genetic polymorphisms *HSD11β1 (rs3753519)* (*p* = 0.00001) and *CRTC2 (rs8450)* (*p* = 0.04) were also associated with a risk of an increase in BMI.

Regarding genotyping of *5-HT2C* (serotonin) *receptor*, Theisen et al. [55] found no association between the *5-HT2C receptor (rs3813929)-759C allele* and weight gain after 12 weeks of clozapine treatment in 97 patients with schizophrenia. Notwithstanding, among patients treated with olanzapine and genotyped for *5-HT2C receptor (rs518147),* Godlewska et al. [62] found that significantly less patients with *-697C* (3/51, *p* ≤ 0.0006) and no patient with *-759T* (0/28, *p* ≤ 0.002) *alleles* experienced a BMI increase ≥10%. In an analysis of body weight change after 4 months of clozapine treatment, Hong et al. [63] showed no relationship with the histamine receptor H1 genotype (rs2067467). The analysis of *DRD2 -141C* *(rs1799732)* by Lencz et al. [64] in patients treated with APs showed that *deletion carriers* gained significantly more weight over time (time-by-genotype interaction, *p* = 0.024). Tiwari et al. [57] showed a nominal association of the CNR1 (rs806378) polymorphism with weight gain in patients treated with clozapine or olanzapine. *T allele* (minor allele) carriers gained more weight (5.96%) than the CC carriers (2.76%, *p* ≤ 0.008), which can be translated into approximately 2.2 kg more weight gain in patients carrying the *T allele* (CC vs. CT + TT, 2.21 ± 4.51 vs. 4.33 ± 3.89 kg; *p* ≤ 0.022). When searching for an association of *COMT Val158Met (rs4680)* variants with MetS, Cote et al. [65] found that atypical AP-treated children with the Met allele had higher systolic (*p* = 0.014) and diastolic (*p* = 0.034) blood pressure, and higher fasting glucose concentrations (*p* = 0.030) compared with children with the Val/Val genotype.

In atypical AP-treated children, Devlin et al. [66] found an association between the *MTHFR* *(rs1801133) 677T allele* with MetS (*p* ≤ 0.05) (OR 5.75 [95% CI 1.18–28.12]). Dong et al. [67] found that the *A2BP1 (rs1478697)* polymorphism was significantly associated with AIWG caused by olanzapine (*p* = 0.0012, Bonferroni corrected *p* = 0.0048). This association was replicated in another sample, including 208 first-episode and drug-naïve patients presenting with schizophrenia after a 4-week treatment with olanzapine (*p* = 0.0092, Bonferroni corrected *p* = 0.0368). Pouget et al. [68] found no association between *TSPO (rs739092, rs5759197, rs138911, rs113515, rs6971, rs6973, rs80411 and rs138926)* polymorphisms and weight change.

**Table 3:** Synthesis of studies investigating metabolic adverse drug reactions.

Study	Diagnosis	Antipsychotic	Dosing	Outcome Measured	Gene Variant	Role of the Genes	Association	Pathophysiology
Devlin et al. (2012)	Not provided	Olanzapine	Not provided	MetS: weight; waist circumference; BMI; DBP and SBP; plasma glucose, insulin, TC; LDL; HDL; TG; ALAT; ASAT	*MTHFR (rs1801133) C677T C;T*	Conversion of folate to 5-methyltetrahydrofolate (active form)	SGA-treated children with T-allele: ↑ prevalence of MetS, ↑ diastolic blood pressure Z-scores, and fasting plasma glucose	Changes in DNA methylation + gene expression profile that favors development of MetS characteristics.
Nussbaum et al. (2014) A	Schizophrenia; BD	Olanzapine	Not provided	Weight gain; BMI; insulin variations	CYP2D6 *4	Drug and steroid metabolism	Patients with the genotype wt/*4, IM have significantly ↑ WG values than the patients without *4 allele.	Nonfunctional *CYP2D6* alleles increase exposure to antipsychotics.
Cote et al. (2015)	Anxiety, Depression, ADHD, Mood disorder, Psychotic disorder, Adjustment disorder, PDD, Other	Olanzapine	Not provided	Cardiometabolic risk factors: Plasma glucose, insulin, TC, LDL, HDL, TG; weight, waist circumference, BMI, DBP and SBP	*COMT Val158Met (rs4680) Met; Val*	Degradation of catecholamines	No significant findings. Interaction observed for SBP z-score. Children with Met allele had ↑ fasting plasma glucose and ↓ HDL	*COMT Val158Met* genotype may influence epigenetic regulation and ↓ activity of COMT = deleterious effect on cardiometabolic dysfunction and BP regulation.
Thümmler et al. (2018)	COS, ASD, ODD (OLZ, CLZ, LOX); COS, PTSD, behavior disorders, ASD, ODD, ID	Olanzapine; Clozapine; Loxapine	Not provided	Lack of therapeutic effect, various ADRs (weight gain, dystonia...)	CYP2D6 *3, *4, *5, *6, *41, Dup	Drug and steroid metabolism	Major adverse events in 4/9 patients	Accumulation of metabolites, *CYP* expression variation with age, drugs which are *CYP* inhibitors
Hong et al. (2002)	schizophrenic disorders	Clozapine	Not provided	Body weight change; BMI	*H1 (rs2067467): Glu, Asp*	H1 (histamine) receptor	No significant correlation between BWC and H1 genotypes.	In animal studies, blocking the H1 receptor = stimulation of feeding behaviors, and ↑ weight gain.
Theisen et al. (2004)	schizophrenia spectrum disorders	Clozapine	mean clozapine dose: 302 ± 128 mg/day (range 100–800 mg/day)	Weight gain; BMI change	*5-HT2CR (rs3813929)-759C/T C,T*	5-HT2CR: serotonin receptor	Higher proportion of patients with the *CC* genotypes with weight gain when compared with those with a *T* allele, this result was not significant.	Serotonin has been suggested to play an important role in the regulation of feeding behavior.
Godlewska et al. (2009)	schizophrenia (mostly paranoid)	Olanzapine	Olanzapine monotherapy: range 20–25mg/day	Weight gain; BMI change	*5-HT2CR (rs3813929) 759C/T C,T;* *5-HT2CR (rs518147) 697G/C G,C*	5-HT2CR: serotonin receptor	A protective effect of *-759T* and *-697C* alleles was found: significantly less patients with *-697C* and no patient with *-759T* alleles experienced body mass index increase above 10%.	Serotonin could play an important role in the regulation of feeding behavior, especially particular through 5-HT2C receptors.
Le Hellard et al. (2009)	schizophrenia spectrum disorders	Clozapine	range 20–25 mg/day	Weight gain; BMI	*44 SNPs: 3 SNPs in INSIG1; 21 SNPs in INSIG2; 3 SNPs in SCAP; 4 SNPs in SREBF1; 13 SNPs in SREBF2*	INSIG1; INSIG2; SCAP; SREBF1 and SREBF2: regulation of biosynthesis and uptake of lipids	Strong association between 3 markers localized within or near the INSIG2 gene (*rs17587100*, *rs10490624* and *rs17047764*) and AIWG.	*SREBP* mediated activation of lipid biosynthesis in cultured cells. *INSIG2* has recently been implicated as a susceptibility gene in obesity
Tiwari et al. (2010)	schizophrenia or schizoaffective disorders	Clozapine, Olanzapine	mean clozapine dose: 285 ± 121 mg/day (range 50–800 mg/day	Weight gain	*20 SNPs in CNR1*	CNR1: cannabinoid receptor	No association of any of the polymorphisms with weight change In the European subgroup, the polymorphism *rs806378* was the only significant SNP in genotypic comparison. Carriers of the *‘T’ allele* gained more weight than the *CC* genotype carriers. In African-Americans a significant association was observed only for *rs1049353* (increased risk for *CT* vs. *CC*).	The *T allele* created a binding site for arylhydrocarbon receptor translocator, a member of the basic helix–loop–helix/Per–Arnt–Sim protein family. Genetic polymorphisms in the *CNR1* gene have been associated with basal metabolic index, obesity and various metabolic parameters.
Lencz et al. (2010)	schizophrenia, schizoaffective or schizophreniform disorder	Olanzapine	Patients randomly assigned to receive either clozapine (500 mg/day), olanzapine (20 mg/day)	Weight gain; BMI change	*DRD2 (rs1799732)* *141C Ins;Del*	DRD2: dopamine receptor	Deletion carriers gained significantly more weight; they began to separate from Ins/Ins homozygotes after 6 weeks of treatment on either medication.	Liability to antipsychotic-induced weight gain may be related to variation in density of D2 receptors.
Jassim et al. (2011)	schizophrenia spectrum disorders	Clozapine	Not provided	Weight gain; BMI change as BMI-1_2 (from the start of the AP until prior to the clozapine administration), Δ BMI-2_3 (during the clozapine administration) and Δ BMI-1_3 (the whole AP treatment) period	*96 SNPs: 13 for ADIPOQ; 10 for FABP3; 7 for PRKAA1; 14 for PRKAA2; 3 for PRKAB1; 4 for PRKAG1; 40 for PRKAG2; 4 for PRKAG3; 1 for FTO*	ADIPOQ; FABP3; PRKAA1; PRKAA2; PRKAB1; PRKAG1; PRKAG2; PRKAG3; FTO: regulation of lipid and energy homeostasis	Allelic and genotypic association between *rs17300539* in the *ADIPOQ* gene and Δ BMI-1_2 and Δ BMI-1_3. 4 other *ADIPOQ* markers showed nominal allelic association to Δ BMI-1_2 (*rs17373414*) or Δ BMI-2_3 (*rs864265*, *rs1501299* and *rs6773957*). *rs6773957* also displayed genotypic association for Δ BMI-2_3, together with *rs3821799*. 1 marker in *PRKAA1* (*rs10074991*) displayed allelic and genotypic association to Δ BMI-1_3. In *PRKAA2*, 3 markers demonstrated weak association either to Δ BMI-1_2 (*rs4912411*) or Δ BMI-1_3 (*rs7519509* and *rs10489617*). In *PRKAG2*, one marker (*rs17714947*) demonstrated allelic, and another marker (*rs7800069*) genotypic association with Δ BMI-2_3.	Adiponectin has recently been suggested as a biomarker for AP-induced metabolic disturbances: negative correlation between circulating levels of adiponectin and BMI, TG and insulin levels in patients taking AP. Variants of AMPK-encoding genes influence the baseline BMI, with limited if any direct effects upon AIWG.
Choong et al. (2013)	Psychotic disorders, mood disorders, others	Clozapine, Olanzapine	Not provided	Weight gain; BMI change	*3 CRTC1 SNPs: rs10402536 G > A; rs8104411 C > T; rs3746266 A >G*	CREB co-activator (mood, memory, energy metabolism...)	Significant association between *CRTC1 rs3746266A > G* and BMI, with G carriers having a lower BMI. After adjustment for the severity of the psychiatric disorder, the association between BMI and *CRTC1 rs3746266A > G* is even stronger. Stronger association in women, especially < 45 years. The *T allele* of *rs6510997C > T* (a proxy of the *rs3746266 G allele*) was associated with lower BMI and fat mass.	Role for the *CRTC1* gene in the regulation of human bodyweight and fat mass consistent with animal models. Psychiatric illness and/or weight gain–inducing psychotropic drugs might play a role in genetically mediated energy homeostasis
Gagliano et al. (2014)	schizophrenia or schizoaffective disorders	Clozapine, Olanzapine	Not provided	Weight gain	*16 PRKAR2B SNPs*	PRKAR2B: regulation of lipid homeostasis	One SNPs *in PRKAR2B (rs9656135)* was significantly associated with AIWG before correcting for multiple testing, but lost significance when adjusting for the 176 effective tests.	Evidence was provided by animal studies suggesting a role of the *PRKAR2B* gene in energy metabolism.
Dong et al. (2015)	schizophrenia	Olanzapine	Not provided	Weight gain; BMI	*4 A2BP1 SNPs: rs10500331, rs4786847, rs8048076, rs1478697, rs10500331*	A2BP1: regulates tissue-specific splicing, involved in neurological function	The SNP *rs1478697* in the *A2BP1* gene was associated with olanzapine-induced WG. The association of *rs8048076* did not remain significant after correction for multiple comparisons.	*A2BP1* gene was preferentially expressed in the human brain; it might affect adiposity via the hypothalamic MC4R pathway, explaining the role of A2BP1 in olanzapine induced AIWG.
Pouget et al. (2015)	schizophrenia of schizoaffective disorders	Clozapine, Olanzapine	Olanzapine dose (mg/d) D: 10.2 ± 2.3 R: 11.8 ± 3.1	Weight gain; lack of therapeutic effect through treatment response (BPRS)	*TSPO 8 SNPs: rs739092, rs5759197, rs138911, rs113515, rs6971, rs6973, rs80411, rs138926*	TSPO: translocator protein, peripheral benzodiazepine receptor	No association between any of the TSPO SNPs and change in overall BPRS. Non significant trend for association between *rs6971* and WG, with an increase in weight for each *Thr* allele an individual carried. In the subset of 78 subjects treated with clozapine or olanzapine, *rs6971* was nominally associated with weight gain, but did not remain significant after multiple testing correction.	Unknown mechanism by which *TSPO* influences glucose lowering and activation of fasting metabolism, possibilities include the altering of steroid synthesis, cytokine production or ROS levels.
Quteineh et al. (2015)	Psychotic disorders, mood disorders, schizoaffective disorders, others	Clozapine, Olanzapine	Not provided	Weight gain, blood pressure and the other components of MetS	*HSD11B1 7 variants: rs12565406 G > T, rs10863782 G > A, rs846910 G > A, rs375319 G > A, rs12086634 T > G, rs4844488 A > G, rs84690 C > T*	HSD11B1: cortisone reductase, reduces cortisone to the active hormone cortisol	Carriers of the variant *rs846910*-A, *rs375319*-A, and *rs4844488-G alleles* showed lower BMI values and lower WC, compared with patients with the wild-type genotypes. Association was exclusively detected in women. For the *rs846906C > T* SNP, only men carrying the *T-allele* showed higher WC compared with noncarriers. Among women, carriers of the *rs846910-A, rs375319-A,* and *rs4844488-G* alleles had lower DBP compared with noncarriers. Among men, carriers of the *T-allele* had higher TG levels compared with noncarriers. Men carrying the *T-allele of rs846906C > T* showed lower HDL-C levels compared with noncarriers.	A direct relationship between aromatase activity and body weight was proposed + estrogen may increase cortisone to cortisol conversion mediated by 11β-HSD1 and cortisol may increase aromatase activity = more estrogen in the tissues. Findings between *rs846906C > T* and lipid traits and BWC in men are not explained.
Saigi et al. (2016)	psychotic disorders, schizoaffective disorders, BD, depression, other	Clozapine, Olanzapine	Not provided	Weight gain, waist circumference, serum lipids, glucose	52 SNPs previously associated with BMI	Weight regulation; glycemia regulation; psychiatric disorders	w-GRS of 32 polymorphisms significantly associated with BMI in men 1 SNP in *CADM2* gene showed a nominal association with BMI over time. At 12 months of treatment, the *rs13078807* polymorphism showed an increase in BMI for each additional risk allele. *HSD11β1 rs3753519* showed an association with lower BMI for *rs3753519* in patients homozygous for the variant allele compared to wild types.	The *HSD11β1* gene codes for a microsomal enzyme-catalyzing tissue regeneration of active cortisol from the inactive form cortisone. It is highly expressed in metabolic tissues such as the liver and adipose tissue. ↑ plasma cortisol levels have been associated with visceral obesity and metabolic syndrome. An overexpression of this gene has been associated with hyperphagia and obesity in mice. CADM2 plays an important role in systemic energy homeostasis.
Menus et al. (2020)	schizophrenia	Clozapine	Clozapine daily dose (mg): 194.3 ± 130.5	Structured questionnaire + BMI, bodyweight (obesity), fasting glucose concentrations, TG, TC, HDL, LDL	CYP1A2 *1C, *1F, *1; CYP3A5 *1, *3; CYP3A4 *1, *1B, *22	Drug and steroid metabolism	No association between *CYP1A2* or *CYP3A4* expression and blood glucose, TG or cholesterol levels in patients. Moderate/high risk obesity was significantly more frequent in low *CYP3A4* expressers. In low *CYP3A4* expressers, a significant correlation was found between clozapine serum concentration (or daily dose) and blood glucose level	The relative activity of *CYP1A2* and *CYP3A4* is assumed to determine which enzyme has a greater role in clozapine metabolism. 5-HT2C antagonism has been reported to be a mechanism underlying atypical AIWG + norclozapine has a greater antagonist effect on 5-HT2C receptors than the parent compound = positive correlation between BMI and norclozapine/clozapine ratios.

OLZ: Olanzapine; CLZ: Clozapine; LOX: Loxapine; PDD: Pervasive Development Disorder; ADHD: Attention Deficit Hyperactivity Disorder; COS: Childhood Onset Schizophrenia. ASD: Autism Spectrum disorder; ODD: Oppositional Defiant Disorder; ID: Intellectual Disability; PTSD: Post-Traumatic Stress Disorder; BD: Bipolar Disorders; SNP: Single-Nucleotide Polymorphism; MetS: Metabolic syndrome; DBP: Diastolic Blood pressure; SBP: Systolic Blood Pressure; SGA: Second-Generation Antipsychotic; IM: Intermediate Metabolizer; BWC: Body Weight Change; AIWG: Antipsychotic-Induced Weight Gain.

#### 3.3.2. Neurological Symptoms: Movement Abnormalities and Seizures

Our query retrieved two studies investigating seizures (28.6%) and five studies addressing movement abnormalities (71.4%), as shown in Table 4. One pediatric and one mixed population study assessed antipsychotic-induced seizures (50%). In addition, two pediatric (40%) and thee mixed studies (60%) investigated movement abnormalities.

Baumann et al. [69] reported an epileptiform seizure, which occurred in a 16-year-old female treated with sertraline and olanzapine. She was found to be CYP3A5 *3/*3 (though, with a preserved CYP3A activity), CYP2B6 *6/*6, and CYP2D6 *4/*4 (PM). Indeed, the resulting high sertraline plasma levels added to the olanzapine treatment could have contributed to the onset of the seizure. Prows et al. [70] found that patients’ combined phenotype (generated via *CYP2C19* and *CYP2D6* phenotypes) was associated with the number of ADRs (*p* = 0.03). Combined *PMs* treated with psychotropics had the highest number of ADRs (among which EPS was classified as a severe ADR), and *combined ultrarapid metabolizers (UMs)* had the lowest number of ADRs. By contrast, Thümmler et al. [3] reported the case of a *CYP2D6 (>2N) UM* 16-year-old male that presented EPS when treated by olanzapine and clozapine. Their case series also mentioned the case of a 14-year-old female, CYP2D6 *4/*41 (PM), who presented numerous ADRs, including EPS, akathisia, and dystonia, when treated with clozapine and loxapine. In patients treated with psychotropic drugs, Vandel et al. [71] observed a higher percentage of carriers of a genotype with *CYP2D6* unfunctional alleles in the group of patients suffering from extrapyramidal ADRs than in the symptom-free patient group (*p* < 0.00001).

Beyond cytochromes, Kohlrausch et al. (2008) [72] found that, in patients treated with clozapine, carriers of the *T825 allele* of the *GNB3 (rs5443)* polymorphism had a higher risk to present a convulsion episode (*p* = 0.007). Ivashchenko et al. [73] observed that patients with *HTR2A (rs6313) C allele* (42.1 vs. 0%, *p* = 0.003), but also patients with *DRD2 (rs1800497) T allele*, more often complained of tremor (50 vs. 21.6%, *p* = 0.039). However, these associations could not be confirmed because of coincidence with higher dosing of antipsychotics. In patients treated with APs, Nicotera et al. [74] found that the COMT Val158Met (rs4680) G/A (Val/Met) genotype was almost exclusively represented in patients presenting with persistent dystonia.

**Table 4:** Synthesis of studies investigating neurological adverse drug reactions.

Study	Diagnosis	Antipsychotic	Dosing	Outcome Measured	Gene Variant	Role of the Genes	Association	Pathophysiology
Baumann et al. (2006)	OCD	Olanzapine	Olanzapine at 2.5 mg/d (day 1) and titrated until 10 mg/d on day 42	Epileptiform seizure	CYP2D6 *4; CYP3A5 *3; CYP2B6 *6; CYP2C9 *1; CYP2C19 *1	Drug and steroid metabolism	*CYP3A5*: PM 100% (but normal CYP3A activity); *CYP2B6*: PM 100% and *CYP2D6*: PM 100% (may explain high sertraline plasma levels)	Seizure favored by high sertraline concentrations + olanzapine
Prows et al. (2009)	Mood disorders; Disruptive behavior; Anxiety, ICD; Psychotic disorders; PDD; ED; Adjustment disorders; Other	Olanzapine	Not provided	Behavioral Intervention Score (BIS); number of PRN doses; LOS; change in GAF from admission to discharge; number of ADRs (sleep disturbances, EPS...)	CYP2D6 *1, *3, *4, *5, Dup; CYP2C19 *1, *2	Drug and steroid metabolism	Significant relationship between combined predicted phenotype and the number of ADRs. Relationship between *CYP2C19*-predicted metabolizing phenotype and number and severity of ADRs.	Increased metabolizing capacity leads to a decrease in drug efficacy and number of ADRs. Regarding *CYP2C19*, its decreased metabolizing ability led to an increase in the number/severity of ADRs
Thümmler et al. (2018)	COS, ASD, ODD (OLZ, CLZ, LOX); COS, PTSD, behavioral disorders, ASD, ODD, ID	Olanzapine; Clozapine; Loxapine	Not provided	Lack of therapeutic effect, various ADRs (EPS, dystonia...)	CYP2D6 *3, *4, *5, *6, *41, Dup	Drug and steroid metabolism	Major adverse events were described in 4/9 patients representing 1/2 of PM and 2/3 of UM.	Accumulation of metabolites + *CYP* expression patterns alter with age + some drugs are inhibitors of *CYP* = might be related to pharmacoresistance.
Ivashchenko et al. (2020)	BPD; schizophrenia; schizoaffective disorder; schizotypal disorder; MDD; delusional disorders	Clozapine; Olanzapine	mean (SGA) (50 [50; 180] mg/day)	Tolerability of psychopharmacology: UKU SERS (salivation, duration of sleep, tremor, akathisia...), SAS, BARS; effectiveness of antipsychotics: PANSS;	CYP2D6 *4, *9, *10; CYP3A4 *22, CYP3A5 *3; *ABCB1 (rs1128503, rs2032582, rs1045642)*; *DRD2 (rs1800497); DRD4 (rs1800955); HTR2A (rs6313)*	*CYP2D6, CYP3A4, CYP3A5*: drug and steroid metabolism; ABCB1: ATP-dependent efflux pump; DRD2 and DRD4: dopamine receptors; HTR2A: serotonin receptor	Patients with *HTR2A rs6313* more often complained of tremor. *DRD2 rs1800497* was significantly associated with tremor.	Associations of *DRD2 rs1800497* and *HTR2A rs6313* with ADEs could not be confirmed because there was coincidence with higher daily doses of antipsychotics.
Vandel et al. (1999)	MDD, dysthymia, OCD, schizophrenia	Olanzapine	Olanzapine 10	EPS (SAS, Leo’s criteria)	CYP2D6 *1A, *2, *2B, *3, *4A, *4D *5, *6B, *9, *10B	Drug and steroid metabolism	Higher % of genotypes with no (extensive) functional alleles in the group of patients suffering from extrapyramidal side effects.	Increased exposure
Kohlrausch et al. (2008)	schizophrenia	Clozapine	Mean daily dose of clozapine: 540.91 mg/day, but varied from 100 to 900 mg/day	Clozapine response (BPRS ↓ 30% = appropriate response); occurrence of clozapine- induced NOGS (clinical interviews)	*GNB3 (rs5443) 825C > T*	GNB3: G-protein (G-protein-coupled receptors GPCRs)	Carriers of the *T825 allele* showed an increased risk for a convulsive episode.	Since dopamine and serotonin receptor subtypes activate intracellular pathways through GPCRs, the effect of the variability in the *GNB3* gene might affect CNS toxicity of clozapine.
Nicotera et al. (2021)	ID, psychotic disorder, schizophrenia spectrum, gait disorder, specific learning disorder, schizotypal personality disorder	Clozapine, Olanzapine	Not provided	Dystonia (review of medical records)	*COMT Val158Met (rs4680) Met; Val COMT L136L (rs4818) G,C*	Degradation of catecholamines	*G/G* and *A/A* genotype polymorphisms of *COMT* gene are associated with a protective effect for developing EPS. *G/A* genotype, almost exclusively present in sensible patients, could be a risk factor for developing dystonia after administration of APs.	The *V158M* polymorphism of the COMT = low enzymatic activity and ↑ dopamine levels in the CNS = this can cause or aggravate EPS in these patients (including parkinsonism, akathisia, dystonia, and dyskinesia).

OCD: Obsessive Compulsive Disorder; ICD: Impulse Control Disorder; PDD: Pervasive Developmental Disorder; ED: Eating Disorder; COS: Childhood onset schizophrenia; ASD: Autism spectrum disorder; ODD: Oppositional Defiant Disorder; PTSD: Post-Traumatic Stress Disorder; ID: Intellectual Disability; BPD: Brief Psychotic Disorder; MDD: Major Depressive Disorder; OLZ: Olanzapine; CLZ: Clozapine; LOX: Loxapine; PRN: Pro re nata, “as needed” basis; LOS: Length of Stay; GAF: Global Assessment of Functioning; UKU SERS: UKU Side Effect Self-Rating Scale; SAS: Simpson-Angus Scale; BARS: Barnes Akathisia Rating Scale; PANSS: Positive And Negative Syndrome Scale; BPRS: Brief Psychiatric Rating Scale; NOGS: New Onset Generalized Seizures; EPS: Extrapyramidal Syndrome; CNS: Central Nervous System.

#### 3.3.3. Lack of Therapeutic Effect

Among studies addressing lack of therapeutic effect (Table 5), pediatric and mixed studies each accounted for a half (4; 50%).

Berel et al. [11] reported four cases of children (1: CYP1A2 *1F/*1F (UM), 2: CYP2D6 *1/*41 (IM) CYP3A5 *1/*1, 3: CYP2C9 *1/*3 (IM), 4: CYP1A2 *1/*1F (UM)) presenting with behavioral disorders of various causes. In all these cases, low clozapine plasma levels led to a lack of therapeutic effect, corrected with fluvoxamine (*CYP1A2* inhibitor) addition. Among children treated with psychotropic drugs, Prows et al. [70] found that the combined phenotype of *CYP2D6* and *CYP2C19* was associated with behavior intervention score (BIS), which is a measure of aggression severity (depending on the number of recorded timeouts/seclusions, therapeutic holds, and physical restraints). In this context, combined PMs had the lowest BIS (highest efficacy), and combined UMs had the highest BIS (lowest efficacy). There was no difference among groups in change in GAF (Global Assessment of Functioning) scores (*p* = 0.90). In children treated with atypical APs, Nussbaum et al. [52] found a significant correlation between the CYP2D6 wt/*4 genotype and higher PANSS (Positive And Negative Syndrome Scale, used in schizophrenia) scores, indicating a poor clinical outcome and a bad response to the atypical antipsychotics (*p* = 0.001). In line with these findings, Thümmler et al. [3] noted that in their case series, five patients out of nine with pharmacoresistant mental health disease presented functional CYP2D6 abnormalities (three patients > 2N (UM), one patient *4/*41 (PM), and one patient *3/*4 (PM)). Conversely, Ivashschenko et al. [73] observed that *CYP2D6*, CYP3A5*3, and *ABCB1* *(rs1128503, rs2032582, rs1045642)* genetic polymorphisms were not significantly associated with a change in the mean score of PANSS between 1 and 14 days of treatment. Yet, the carriers of *DRD2 C2137T (rs1800497)* had a higher degree of the PANSS “productive symptoms” subscale score change (M = −7.5 (−9; −4.5) vs. M = −4 (−7; −2), *p* = 0.005). In addition, for *HTR2A T102C (rs6313)* polymorphism, the improvement of *C-allele* carriers in PANSS subscale “negative symptoms” was significantly lower than in *TT homozygotes* (M = −1 (−3.25; 0.25) vs. M = −3 (−6; −1), *p* = 0.037, respectively).

Regarding other genes, Kohlrausch et al. (2008) [72] found an increased frequency of homozygosity for the *GNB3 (rs5443) T825* allele among non-responders to clozapine (*p* = 0.021). In 2010, Kohlrausch et al. [75] found significant differences between responders and non-responders to clozapine involving the *5-HTT HTTLPR (rs25531)* polymorphism. Non-responders displayed a higher frequency of *S’-allele* (*p* = 0.01) and were more likely to *be S’/S’ homozygous* or *S’/L’ heterozygous* than the responders (*p* = 0.04). In patients treated with APs, Pouget et al. [68] found no association between investigated SNPs for TSPO *(rs739092, rs5759197, rs138911, rs113515, rs6971, rs6973, rs80411,* and *rs138926)* and change in Brief Psychiatric Rating Scale (BPRS) (all *p*_uncor_ > 0.05).

**Figure 2:**
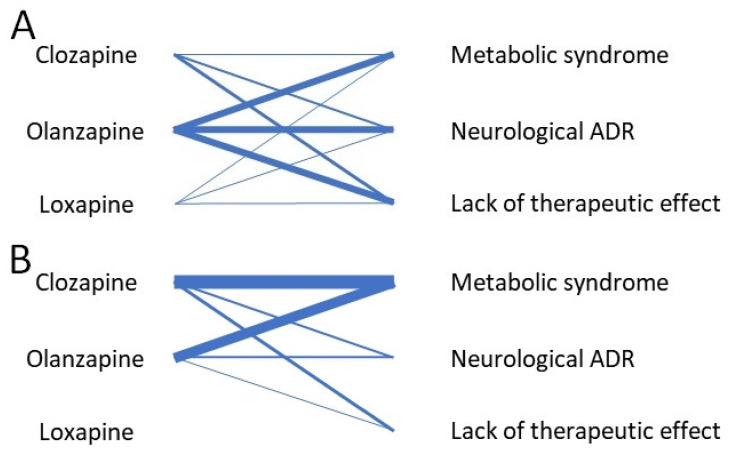
(**A**) Network diagram for pediatric pharmacogenetic studies regarding CYP1A2-metabolized AP and their adverse drug reactions. (**B**) Network diagram for mixed population pharmacogenetic studies regarding CYP1A2-metabolized AP and their adverse drug reactions. The thickness of the connecting lines corresponds to the number of studies evaluating the drug–ADR association.

Figure 2A,B summarizes the number of studies evaluating the drug–ADR association, for pediatric and mixed population studies, respectively.

**Table 5:** Synthesis of studies investigating lack of therapeutic effect.

Study	Diagnosis	Antipsychotic	Dosing	Outcome Measured	Gene Variant	Role of the Genes	Association	Pathophysiology
Prows et al. (2009)	Mood disorders; Disruptive behavior; Anxiety, ICD; Psychotic disorders; PDD; ED; Adjustment disorders; Other	Olanzapine	Not provided	Behavioral Intervention Score (BIS); number of PRN doses; LOS; change in GAF from admission to discharge; number of ADRs (including sleep disturbances, EPS...)	CYP2D6 *1, *3, *4, *5, Dup; CYP2C19 *1, *2	Drug and steroid metabolism	C-PM group had lower BIS (higher efficacy), C-UM group had highest BIS (lowest efficacy). Significant relationship between combined predicted phenotype and the number of ADRs. Relationship between *CYP2C19*-predicted metabolizing phenotype and number and severity of ADRs.	Increased metabolizing --> decrease in drug efficacy and number of ADRs. *CYP2C19*′s decreased metabolizing ability --> ↑ in the number/severity of ADRs
Nussbaum et al. (2014) B	Schizophrenia; BD	Olanzapine	Not provided	Lack of therapeutic effect: change in PANSS	CYP2D6 *4	Drug and steroid metabolism	Significant correlations between wt/*4 genotype, ↑ PANSS scores, a poor clinical outcome and a bad drug response	Drug response to atypical APs correlated with the *CYP2D6* genotype
Thümmler et al. (2018)	COS, ASD, ODD (OLZ, CLZ, LOX); COS, PTSD, behavioral disorders, ASD, ODD, ID	Olanzapine; Clozapine; Loxapine	Not provided	Lack of therapeutic effect, various ADRs (weight gain, dystonia...)	CYP2D6 *3, *4, *5, *6, *41, Dup	Drug and steroid metabolism	5/9 patients with pharmacoresistant mental health disease presented functional *CYP2D6* abnormalities.	*CYP* expression patterns varies with age, in addition to direct metabolism by *CYP2D6*, some drugs are inhibitors of *CYPs*
Ivashchenko et al. (2020)	BPD; schizophrenia; schizoaffective disorder; schizotypal disorder; MDD; delusional disorders	Clozapine; Olanzapine	mean (SGA) (50 [50; 180] mg/day)	Tolerability of psychopharmacology: UKU SERS, SAS, BARS; effectiveness of antipsychotics: PANSS; salivation, duration of sleep, tremor, akathisia...	CYP2D6 *4, *9, *10; CYP3A4 *22, CYP3A5 *3; *ABCB1 (rs1128503, rs2032582, rs1045642)*; *DRD2 (rs1800497); DRD4 (rs1800955); HTR2A (rs6313)*	CYP2D6, CYP3A4, CYP3A5: drug and steroid metabolism; ABCB1: ATP-dependent efflux pump; DRD2 and DRD4: dopamine receptors; HTR2A: serotonin receptor	Carriers of *DRD2 C2137T (rs1800497)* had higher degree of productive symptoms subscale score change. Significant associations between the *HTR2A T102C* polymorphism (*rs6313*) and the subscale negative symptoms: the improvement in *C-allele* carriers significantly lower than in TT homozygotes.	*DRD2 rs1800497 T-allele* is associated with ↓ activity of D2 receptors (↓ binding to the ligand). ↓ in HTR2A expression in CNS may alter antipsychotics’ effect in terms of reducing negative symptoms.
Berel et al. (2021)	Tourette syndrome and ID; behavioral disorders and neurodevelopmental delay; EOS; ASD with catatonia	Clozapine	clozapine dosage (500 mg/day); clozapine dosage (300 mg/day); clozapine dosage between 400 and 500 mg/day; clozapine dosage (200 mg/day)	Clozapine plasma levels and clinical improvement (SAPS, ABC) with adjunction of fluvoxamine	CYP1A2 *1F, *1; CYP2D6 *1, *4, *10, *41; CYP2C19 *1, *2; CYP3A5 *1, *3; CYP3A4 *1; CYP2C9 *1, *3	Drug and steroid metabolism	*CYP1A2* UM: low clozapine plasma levels, ↑ with fluvoxamine addition (clinical improvement) *CYP2D6* IM; *CYP3A5* UM: low clozapine plasma levels --> fluvoxamine addition clozapine levels ↑ (clinical improvement) *CYP2C9* IM: low clozapine plasma levels, ↑ with fluvoxamine addition (clinical improvement) *CYP1A2* UM *CYP2D6* IM *CYP2C19* IM: low clozapine plasma levels, ↑ with fluvoxamine addition (clinical improvement)	Genotypes explaining low clozapine plasma level + lack of improvement with previous treatments
Kohlrausch et al. (2008)	schizophrenia	Clozapine	Mean daily dose of clozapine: 540.91 mg/day, but varied from 100 to 900 mg/day	Clozapine response (BPRS, reduction 30% = appropriate response); occurrence of clozapine induced new onset generalized seizures (clinical interviews)	*GNB3 (rs5443) 825C > T*	GNB3: G-protein (G-protein-coupled receptors GPCRs)	Homozygosis for the *T825 allele* more frequent among NR Homozygosis for the *C825 allele* more frequent among responders.	Dopamine and serotonin receptor subtypes activate intracellular pathways through GPCRs, the variability in *GNB3* gene might affect medication response.
Kohlrausch et al. (2010)	schizophrenia	Clozapine	Patients received clozapine at doses ranging from 100 to 900 mg daily; mean daily dose of clozapine: 540.91 mg/day.	Lack of therapeutic effect: non responders/responders (30% reduction BPRS)	*5-HTT HTTLPR (rs25531) LA, LG, S; VNTR Stin2 9, 10, 12 repeats*	5-HTT: serotonin transporter	The *S’-allele* of *HTTLPR/rs25531* was more frequent in NR. No significant association between the polymorphisms of *VNTR Stin2* and clozapine response.	Carriers of the low expression *allele S’* would be under increased risk for poor response to clozapine, through the influence in availability of extracellular serotonin concentrations at all synapses. Since the action of clozapine is by antagonism of serotonin receptors, the serotonin transporter coded by the *L’L’* genotype (higher expression compared with the *S’ allele*), mediates more active re-uptake of serotonin --> less serotonin would be available to compete with clozapine for the serotonin receptors, facilitating its action.
Pouget et al. (2015)	schizophrenia of schizoaffective disorders	Clozapine, Olanzapine	Not provided	Weight gain; lack of therapeutic effect through treatment response (BPRS)	*TSPO 8 SNPs: rs739092, rs5759197, rs138911, rs113515, rs6971, rs6973, rs80411, rs138926*	TSPO: translocator protein, peripheral benzodiazepine receptor	We found no association between any of the *TSPO* SNPs and change in overall BPRS. Nonsignificant trend for association between *rs6971* and weight gain, with an increase in weight for each *Thr allele* an individual carried. In the subset of 78 subjects treated with clozapine or olanzapine, *rs6971* was nominally associated with weight gain, but did not remain significant after multiple testing correction.	*TSPO* may act as a modifier gene, affecting clinical features of schizophrenia not investigated in the study. Although the mechanism by which *TSPO* influences glucose lowering and activation of fasting metabolism is unknown, possibilities include the altering of steroid synthesis, cytokine production or ROS levels.

ICD: Impulse Control Disorder; PDD: Pervasive Developmental Disorder; ED: Eating Disorder; COS: Childhood Onset Schizophrenia; ASD: Autism spectrum disorder; ODD: Oppositional Defiant Disorder; PTSD: Post-Traumatic Stress Disorder; ID: Intellectual Disability; BPD: Brief Psychotic Disorder; MDD: Major Depressive Disorder; OLZ: Olanzapine; CLZ: Clozapine; LOX: Loxapine; PRN: Pro re nata, “as needed” basis; LOS: Length of Stay; GAF: Global Assessment of Functioning; UKU SERS: UKU Side Effect Self-Rating Scale; SAS: Simpson-Angus Scale; BARS: Barnes Akathisia Rating Scale; PANSS: Positive and Negative Syndrome Scale; BPRS: Brief Psychiatric Rating Scale; ABC: Aberrant Behavior Checklist; SAPS: Scale for the Assessment of Positive Symptoms; EPS: Extrapyramidal Syndrome.

#### 3.3.4. Others

Studies investigating other ADRs were represented by a majority of pediatric studies (5; 62.8%), the remaining 3 (37.5%) relying on mixed-population samples.

Butwicka et al. [76] reported the case of a patient who presented a neuroleptic malignant syndrome when treated with olanzapine. His *CYP2D6* genotype was CYP2D6*4/*4 (PM), indicating a lack of activity. Likewise, Thümmler et al. mentioned the case of a *CYP2D6 (>2N)* (UM) adolescent presenting a clozapine-induced hepatic cytolysis. They also reported a case of a CYP2D6 *4/*41 (PM) adolescent with, among other ADRs, galactorrhea and constipation, treated with clozapine and loxapine. In patients treated with atypical APs, Grădinaru et al. [77] found that the mean level of prolactin was higher for IMs than for extensive (normal) metabolizers (EMs) at each time point except baseline. Menus et al. [61] noted a significant effect of *CYP3A4* expression on constipation (47.1% in normal/high *CYP3A4* expressers, 71.4% in low *CYP3A4* expressers, OR = 3.6 (95% CI = 0.9–14.1), *p* = 0.06). Ivashschenko et al. [73] found a significantly more frequent increased dream activity in *CYP2D6* IMs compared to EMs (54 vs. 22%, *p* = 0.043). Increased duration of sleep was more frequent among *TT homozygotes* of *ABCB1* (*rs2032582*) polymorphism (50 vs. 15.8%, *p* = 0.006) and *TT* of *ABCB1* (*rs1045642*) polymorphism (41.7 vs. 8.2%, *p* = 0.007). *DRD2 (rs1800497) T allele* was significantly associated with constipation (25 vs. 5.4%, *p* = 0.039). 

Beyond cytochromes assessments, Mosyagin et al. [78] studied a population of schizophrenic patients having presented a drug-induced agranulocytosis. They found that for *MPO* *(rs2333227)* polymorphism, the *AA carriers* (low activity) were overrepresented among cases (OR = 4.16 (95% CI 0.86–20.3), *p* = 0.056). This finding was even more marked in clozapine-induced agranulocytosis (*p* = 0.04). Ocete-Hita et al. [79] investigated idiosyncratic Drug-Induced Liver Injury (DILI) in a pediatric sample, in which one case has been imputed to olanzapine. The human leucocyte antigens HLA-DRB*12 (OR = 9.3 (95% CI 1–88.1), *p* = 0.05) and HLA-DQA*0102 (OR = 2.51 (95% CI 0.9–6.5), *p* = 0.058) were more commonly found in children presenting DILI. Using the Penn Conditional Exclusion Test (PCET), Nelson et al. [80] investigated the relationship of performance errors (as a reflection of cognitive flexibility alteration) with *COMT Val158Met (rs4680)* genotype in patients treated with atypical APs. *Met carriers* displayed significant changes for error type (F(1,62) = 14.874, *p* < 0.001) and time (F(1,62) = 14.068, *p* < 0.001), characterized by a decrease in perseverative and regressive errors following AP treatment. Among the *Val homozygotes*, the perseverative error rate was not modified after treatment, while regressive errors rate increased (F(1,36) = 6.26, *p* = 0.017).

### 3.4. Main Implications of Cytochromes Genotyping

Among studies involving cytochrome genotyping, nine relied on exclusively pediatric samples (81.8%), while two (18.2%) were based on mixed populations. Most of the studies (10; 90.9%) investigating a potential cytochrome involvement were genotyping at least one *CYP2D6* genetic polymorphism. Then, *CYP3A5* genetic polymorphisms were assessed in four studies (36.3%), followed by *CYP2C19* and *CYP3A4* (3; 27.2%), *CYP2C9* and *CYP1A2* (2; 18.1%), and *CYP2B6* (1; 9.1%).

Vandel et al. [71] showed a higher percentage of genotypes, including at least one allele characterized by an extensive enzyme metabolic capacity for *CYP2D6* in the symptom-free group (86%) in comparison with 45.4% in the group suffering from EPS. The genotypes deprived from extensive functional alleles were more frequent (54.4%) in the group of patients suffering from EPS than in the other group (14%).

Butwicka et al. [76] reported the case of a 16-year-old male who experienced a neuroleptic malignant syndrome while being treated by olanzapine. This patient displayed a CYP2D6 *4/*4 (PM) genotype, leading to a decreased *CYP2D6* activity. Nussbaum et al. [51] found that patients showing a CYP2D6 wt/*4 genotype presented a higher BMI than patients showing a *wt/wt* genotype. A difference across these groups was also noted for insulin values. Nussbaum et al. [52] further noted that the PANSS score in the CYP2D6 wt/*4 group was higher than in the *wt/wt* group. Indeed, the first patients would have exhibited no adequate drug response.

As stated above, Thümmler et al. [3] described five young patients with pharmacoresistant mental health disease who displayed *CYP2D6* abnormalities: three patients were *>2N* UM and two patients were PM with *4/*41 and *3/*4 polymorphisms. Major psychotropic ADRs were found in four patients (EPS, akathisia, dystonia, binge eating and weight gain, hepatic cytolysis, galactorrhea, and constipation inter alia).

Grădinaru et al. [77] found that, in *CYP2D6* poor and intermediate metabolizers, the use of atypical APs led to a significant increase in prolactin levels from baseline to 18 months. In IMs, the mean level of prolactin was higher than in EMs at each time point except baseline. After 6 months of AP treatment, IMs displayed a significant increase in prolactin level, over EMs.

Ivashschenko et al. [73] noted an increased dream activity in *CYP2D6* IMs compared to NMs (54 vs. 22%; *p* = 0.043). *CYP2D6* was not significantly associated with a change in the mean score of the PANSS between 1 and 14 days of treatment.

Prows et al. [70] found a relationship between *CYP2D6*-predicted metabolizing phenotype and BIS (*p* = 0.01). Indeed, they noted a statistically significant relationship between combined phenotype (*CYP2D6* and *CYP2C19*) and BIS (*p* = 0.01).

In the case series of Berel et al. [11], the second patient presented a *CYP2D6* IM phenotype and a CYP3A5 *1/*1 polymorphism, and these profiles could have contributed to previous high aripiprazole and low haloperidol plasma levels.

In Ivashschenko et al.’s study [73], CYP3A5*3 polymorphism was not significantly associated with changes in the mean score of the PANSS between 1 and 14 days of treatment.

In Prows et al.’s study [70], while a significant association between combined phenotype (*CYP2D6* and *CYP2C19*) and BIS was found, no relationship was detected between *CYP2C19*-predicted metabolizing phenotype and BIS (*p* = 0.57). Nonetheless, a relationship between *CYP2C19*-predicted metabolizing phenotype and the number of ADRs was observed (*p* = 0.01). *CYP2C19*-predicted metabolizing phenotype has also been linked to the type of ADRs (severe vs. mild vs. none, *p* = 0.04).

In the study of Menus et al. [61], exaggerated clozapine concentrations (>600 ng/mL) were more frequently noted in low *CYP3A4* expressers (22%) than in normal/high expressers (2.7%) (low vs. normal/high expressers: OR = 9.8 (95% CI 1.8–55.0), *p* = 0.009). They also noted an association between norclozapine formation and *CYP3A4* expression (0.56 ± 0.17 vs. 0.98 ± 0.62, *p* < 0.0001). However, no association was found between *CYP3A4* expression and blood glucose, TG, or cholesterol (total, HDL, and LDL) levels in patients (*p* > 0.1). Still, moderate/high risk obesity was significantly more frequent in low *CYP3A4* expressers than in normal expressers (13.6% of *CYP3A4* low expressers, 1.5% of *CYP3A4* normal/high expressers, OR = 13.5 (95% CI 1.2–147.9), *p* = 0.045). *CYP3A4* low expressers more frequently reported constipation, as stated before. In low *CYP3A4* expressers only, significant correlations were found between clozapine serum concentration and blood glucose level (r = 0.52, *p* = 0.02), and between glucose concentrations and the daily dose of clozapine (r = 0.49, *p* = 0.03). In normal/high *CYP3A4* expressers, fasting glucose (r = 0.27, *p* = 0.03) and TG levels (r = 0.26, *p* = 0.048) significantly correlated with norclozapine/clozapine ratios.

In the study of Berel et al. [11], the third patient was found to display a CYP2C9*1/*3 heterozygous genotype. Leading to a *CYP2C9* IM phenotype, it could partly explain the low clozapine plasma levels.

Berel et al. [11] reported in their case series two 11-year-old patients with low clozapine plasma levels, which were found to be *CYP1A2* UM (CYP1A2*1F/*1F and CYP1A2*1/*1F, respectively). Therefore, this issue has been corrected by the adjunction of fluvoxamine, a potent *CYP1A2* inhibitor. Menus et al. [61] demonstrated a contribution of *CYP1A2* to norclozapine production (0.86 ± 0.55 vs. 1.17 ± 0.70, *p* = 0.0007). Yet, no association was found between *CYP1A2* expression and blood glucose, TG, or cholesterol (total, HDL, and LDL) levels in patients (*p* > 0.1). Similarly, *CYP1A2* expression has not been linked with obesity (*p* > 0.1). None of the ADRs reported by patients was influenced by their *CYP1A2* expression (*p* > 0.1).

In the case report of Baumann et al. [69], CYP2B6 *6/*6 homozygosity added to a PM *CYP2D6* phenotype and to an olanzapine co-prescription, may have favored the occurrence of the epileptiform seizure.

## 4. Discussion

Our review aimed to assess whether pharmacogenetic mechanisms underly the occurrence of olanzapine, clozapine, and loxapine ADRs in children and youth. Several included publications investigated the genes involved in neurotransmission (*COMT* [65,74,80], serotonin receptors/transporters [55,62,73], dopamine receptors [64,73]), and in energy and lipid homeostasis (AMP-K related genes [54,56], *HSD11β1* [58,59]), mostly regarding weight gain (or MetS). However, findings regarding possible associations were sometimes conflicting. While *COMT Val158Met (rs4680)* genetic polymorphism may have influenced epigenetic regulation and, therefore, decreased activity of COMT, contributing to a deleterious effect in adults [81], Cote et al. [65] found no significant association in children. Whereas Theisen et al. [55] retrieved no association between the *5-HT2C receptor gene (rs3813929)* polymorphism and clozapine-induced weight gain, Godlewska et al. [62] found a protective effect of -759T and -697C alleles. In antipsychotic-naive patients, Houston et al. [82] did not find similar associations. However, highlighting the possible association of *DRD2* polymorphisms with increased weight gain, their findings supported Lencz et al.’s [64] conclusions. Otherwise, while our query yielded one study addressing the role of *HLA* gene variations in DILI (Ocete-Hita et al.) [79], we did not retrieve similar approaches regarding clozapine-induced neutropenia and agranulocytosis that formerly have been investigated [83].

Cytochromes genotyping (and phenotyping) was the preferred approach when investigating ADRs, especially in pediatric studies. Studies relying on large sample size underlined increased weight gain [51], prolactin levels [77], risk of EPS [71], and impaired treatment response [52] in patients deprived from at least one functional allele for *CYP2D6*, resulting in increased drug exposure. While the findings regarding movement abnormalities and lack of therapeutic effect concur with existing evidence [84,85], AIWG [86] and hyperprolactinemia [87] were not consistently linked with *CYP2D6* impairments. However, olanzapine is mostly metabolized by *CYP1A2* (and to a lesser extent by *CYP2D6* and *CYP3A4*) [88,89], clozapine is mainly metabolized by *CYP3A4* and *CYP1A2* (with *CYP2D6* playing a minor role) [16,90], and loxapine is primarily metabolized by *CYP1A2* (then by *CYP3A4* and *CYP2D6*) [19]. Despite the fact that Menus et al. [61] found no association between *CYP1A2* expression and any ADR, some variants have been formerly linked to tardive dyskinesia [91,92] and to an increased risk of insulin and lipid elevation [93].

Indeed, some of these discrepancies may originate from several limitations of the evidence included in our review. First, we chose to focus on studies involving children and youth, often characterized by smaller samples and thus lack of power to show an existing difference, and lower-evidence study designs (case reports/series). Several large cohorts were (at least partially) overlapping, therefore lowering the total size of the investigated population. Second, we aimed to assess the pharmacogenetic causes of ADRs related to olanzapine, clozapine, and loxapine, whereas several of our largest sample size studies investigated atypical APs indiscriminately. Furthermore, Thümmler et al. [3] only reported a case of patients treated with loxapine, which may be due to French-specific prescription behaviors [23,24]. Third, apart from metabolic changes, ADRs were subject to heterogeneous outcome measurements (EPS, clinical improvement), which may have prevented us from direct comparisons between different studies. Fourth, most studies lacked consideration for potential interacting factors with AP-induced side effects, such as co-treatments, inflammation, weight change, dietary habits, smoking, and/or consumption of caffeine. These factors may be prevailing, especially in transitioning-age youths, and are important to consider. Fifth, our quality assessment of the studies (see Methods), relying on a tool adapted from the checklist by Jorgensen and Williamson [50], yielded an average score of 11.3/24. Overall, some issues of concern were the lack of information upon quality control methods, handling of missing data, and population stratification. In studies including children and youth only, lack of adjustments for multiple testing and of HWE testing were frequent additional flaws, therefore lowering the mean quality score of these studies (9.1/24). Furthermore, the quality assessment tool we relied on may be used as a checklist for further pharmacogenetic studies, to improve the comprehensiveness of the presented results.

In fact, in addition to proper pediatric studies, and considering the foreseeable scarce body of evidence among this population, we accepted to include studies involving at least one youth patient (see Methods) [44]. Thus, while broadening the study population, it may have lowered the impact of the children’s metabolic characteristics. As stated above, the features of the included studies did not permit a strict comparison, preventing any meta-analysis. Nevertheless, our grouping strategy, relying on the main ADR classes (see Methods), enabled qualitative assessments. As a flaw inherent to systematic reviews, reporting bias limits the interpretation of our findings, even if several studies showed negative results. Furthermore, as the overall quality of evidence could not be estimated with reference methods such as GRADE [94], the methodological quality of our included pharmacogenetic studies was assessed via a tool adapted from the checklist of Jorgensen and Williamson [50] (see Methods). Then, a quality assessment was conducted among pediatric and mixed-population studies, allowing us to detect the main issues of concern in each study category. For each database query, the two screening steps and the quality scoring were subject to a dual assessment (D.M. and A.O.G.), which may have limited sources of bias.

While findings in children and youth pharmacogenetics are conflicting regarding olanzapine, clozapine, and loxapine, the benefits of genotyping in clinical use may be limited by lack of sufficient evidence, the barriers to routine use, and overall impact [95]. However, the dose–effect relationship is significantly influenced by cytochromes, holding sway over exposure to the medication [96]. Yet, in comparison with *CYP2D6*, *CYP1A2* remains less investigated, while olanzapine and clozapine’s ADRs are serious. Furthermore, cases of major clinical improvement were fostered by *CYP1A2* genotyping [11], although its benefit is not collective yet. The use of advanced technologies, such as WGS, might provide an interesting complement, broadening the research spectrum in psychiatric disorders [40,41]. From this perspective, further studies addressing the cytochromes’ and other genes’ (involved in energy homeostasis, metabolism, neurotransmission *inter alia*) impact should consider potential polypharmacy and intercurrent modifications in the metabolism of children and youth. Further studies may provide insights into possible cross-talks between the pathways associated with ADRs and GABA-A signaling, identifying new drug targets and therefore paving the way for the development of new antipsychotic drugs with variable receptor affinities. These drugs could constitute alternatives to thienobenzodiazepines, dibenzodiazepines, and dibenzoxazepines, and improve the acceptability of treatments. Phenotypical variations due to ancestry and/or infrequent cytochrome variants should also be taken into account by studying larger pediatric samples that originate from different countries. Determined by genetics, but influenced by the environment, *CYP1A2* and its interactions should be further investigated, to improve assessment of the risk–benefit balance in children and youth treated with olanzapine, clozapine, and loxapine.

## Data Availability

Data sharing not applicable.

## Supplementary Materials

The following supporting information can be downloaded at: https://www.mdpi.com/article/10.3390/ph15060749/s1, Table S1 Quality assessment of included pediatric studies; Table S2 Quality assessment of included mixed population studies.
