# Supplementary material for: Adverse Drug Reactions of Olanzapine, Clozapine and Loxapine in Children and Youth: A Systematic Pharmacogenetic Review
Source: Pharmaceuticals (Basel). 2022 Jun 14;15(6):749. doi: 10.3390/ph15060749 (PMC9230864; doi:10.3390/ph15060749)
Supplement: Supplementary file 1 [file pharmaceuticals-15-00749-s001.zip › pharmaceuticals-1723646-supplementary.pdf]

Table S1. Quality assessment of included pediatric studies (n = 12)

Study	1. Choosing the genes/SNPs to genotype				2. Sample Size			3. Study Design
	Was a literature review undertaken and the findings summarized?	Are reasons given for choosing the genes and SNPs genotyped?	Is method to adjust for multiple testing described?	Are precise p-values provided for all associations?	What is the sample size?	Are details given of how the sample size was calculated?	Are details given of the a priori power to detect effect sizes of varying degrees?	What is the study design?
Baumann et al. (2006)	YES	YES	NO	NO	1	NO	NO	Case Report
Prows et al. (2009)	YES	YES	NO	YES	279	NO	NO	Cohort study
Devlin et al. (2012)	YES	YES	NO	YES	105	NO	NO	Case-control study
Nussbaum et al. (2014) a	YES	YES	NO	NO	81	NO	NO	Cohort study
Nussbaum et al. (2014) b	YES	YES	NO	NO	81	NO	NO	Cohort study
Butwicka et al. (2014)	YES	YES	NO	NO	1	NO	NO	Case Report
Cote et al. (2015)	YES	YES	YES	YES	134	NO	NO	Case-control study
Ocete-Hita et al. (2017)	YES	YES	YES	YES	92	NO	NO	Case-control study
Thümmler et al. (2018)	YES	YES	NO	NO	9	NO	NO	Case series
Grădinaru et al. (2019)	YES	YES	NO	YES	81	NO	NO	Cohort study
Ivashchenko et al. (2020)	YES	YES	YES	YES	53	NO	NO	Cohort study
Berel et al. (2021)	YES	YES	NO	NO	4	NO	NO	Case series

Table S1. Quality assessment of included pediatric studies (n = 12) – continued.

Study	4. Reliability of Genotypes				5. Missing Genotype Data					
	Is the genotyping procedure described?	Are the primers described?	Were quality control methods used and described?	Are any genotype frequencies previously reported quoted?	Were genotyping personnel blinded to outcome status?	Is extent of missing data summarized?	Where extent is summarized are reasons for missing data given?	Are checks for missingness at random reported?	Is missing genotype data imputed?	Does paper quote number of patients contributing to each analysis?
Baumann et al. (2006)	YES	NO	NO	NO	NO	NO	N/A	N/A	N/A	YES
Prows et al. (2009)	YES	NO	NO	NO	NO	NO	N/A	N/A	N/A	YES
Devlin et al. (2012)	YES	NO	NO	YES	NO	NO	N/A	N/A	N/A	YES
Nussbaum et al. (2014) a	YES	NO	NO	YES	NO	NO	N/A	N/A	N/A	YES
Nussbaum et al. (2014) b	YES	NO	NO	NO	NO	NO	N/A	N/A	N/A	YES
Butwicka et al. (2014)	YES	NO	NO	NO	NO	NO	N/A	N/A	N/A	YES
Cote et al. (2015)	YES	NO	NO	NO	NO	NO	N/A	N/A	N/A	YES
Ocete-Hita et al. (2017)	YES	NO	NO	NO	NO	NO	N/A	N/A	N/A	YES
Thümmler et al. (2018)	YES	NO	NO	YES	NO	NO	N/A	N/A	N/A	YES
Grădinaru et al. (2019)	YES	NO	NO	YES	NO	NO	N/A	N/A	N/A	YES
Ivashchenko et al. (2020)	YES	NO	NO	NO	NO	YES	NO	N/A	N/A	YES
Berel et al. (2021)	NO	NO	NO	NO	NO	NO	N/A	N/A	N/A	YES

Table S1. Quality assessment of included pediatric studies (n = 12) - continued.

Study	6. Population Stratification			7. Hardy-Weinberg Equilibrium (HWE)		8. Choice and Definition of Outcomes			Score
	If paper does quote number of patients contributing to analyses does this agree to sample size?	Are tests undertaken for cryptic population stratification?	Is cryptic population stratification adjusted for in the analyses?	Was HWE tested?	Where test undertaken, are SNPs deviating from HWE (or their absence) highlighted and excluded from further analysis?	Does the paper clearly define all outcomes investigated?	Is justification provided for the choice of outcomes?	Are results shown for all outcomes mentioned?	
Baumann et al. (2006)	YES	NO	N/A	NO	N/A	YES	YES	YES	8
Prows et al. (2009)	YES	NO	N/A	NO	N/A	YES	YES	YES	9
Devlin et al. (2012)	NO	NO	N/A	NO	N/A	YES	YES	YES	9
Nussbaum et al. (2014) a	YES	NO	N/A	NO	N/A	YES	YES	YES	9
Nussbaum et al. (2014) b	YES	NO	N/A	NO	N/A	YES	YES	YES	8
Butwicka et al. (2014)	YES	NO	N/A	NO	N/A	YES	YES	YES	8
Cote et al. (2015)	YES	NO	N/A	NO	N/A	YES	YES	YES	10
Ocete-Hita et al. (2017)	YES	NO	NO	NO	N/A	YES	YES	YES	10
Thümmler et al. (2018)	YES	NO	N/A	NO	N/A	YES	YES	YES	9
Grădinaru et al. (2019)	YES	NO	N/A	NO	N/A	YES	YES	YES	10
Ivashchenko et al. (2020)	YES	NO	N/A	YES	YES	YES	YES	YES	13
Berel et al. (2021)	NO	NO	N/A	NO	N/A	YES	YES	YES	6

NO = Not mentioned in the manuscript or in the Method(s) paper that the authors referenced. N/A = Not Applicable as the answer to the main question is "No".

The assessment tool was adapted from Jorgensen and Williamson (2008).

Table S2. Quality assessment of included mixed population studies (n = 20)

Study	1. Choosing the genes/SNPs to genotype				2. Sample Size			3. Study Design
	Was a literature review undertaken and the findings summarized?	Are reasons given for choosing the genes and SNPs genotyped?	Is method to adjust for multiple testing described?	Are precise p-values provided for all associations?	What is the sample size?	Are details given of how the sample size was calculated?	Are details given of the a priori power to detect effect sizes of varying degrees?	What is the study design?
Vandel et al. (1999)	YES	YES	NO	NO	65	NO	NO	Case-control study
Hong et al. (2002)	YES	YES	YES	YES	88	NO	NO	Cohort study
Mosyagin et al. (2004)	YES	YES	NO	YES	159	NO	NO	Case-control study
Theisen et al. (2004)	YES	YES	NO	YES	97	NO	NO	Cohort study
Kohlrausch et al. (2008)	YES	YES	NO	YES	121	NO	YES	Cohort study
Godlewska et al. (2009)	YES	YES	YES	YES	107	NO	NO	Cohort study
Le Hellard et al. (2009)	YES	YES	YES	YES	160	NO	NO	Cohort study
Tiwari et al. (2010)	YES	YES	YES	YES	183	NO	YES	Cohort study
Lencz et al. (2010)	YES	YES	NO	YES	58	NO	NO	Cohort study
Kohlrausch et al. (2010)	YES	YES	NO	YES	116	NO	NO	Cohort study
Jassim et al. (2011)	YES	YES	NO	YES	160	NO	NO	Cohort study
Choong et al. (2013)	YES	YES	YES	YES	444	NO	NO	Cohort study
Gagliano et al. (2014)	YES	YES	YES	YES	99	YES	YES	Cohort study
Dong et al. (2015)	YES	YES	YES	YES	536	NO	YES	Cohort study
Pouget et al. (2015)	YES	YES	YES	YES	1445	NO	YES	Case-control study
Quteineh et al. (2015)	YES	YES	YES	YES	834	NO	NO	Cohort study
Saigi et al. (2016)	YES	YES	YES	YES	750	NO	NO	Cohort study
Nelson et al. (2018)	YES	YES	NO	YES	71	NO	NO	Case-control study
Menus et al. (2020)	YES	YES	NO	YES	96	NO	NO	Cohort study
Nicotera et al. (2021)	YES	YES	NO	YES	21	NO	NO	Case-control study

Table S2. Quality assessment of included mixed population studies (n = 20)

Study	4. Reliability of Genotypes					5. Missing Genotype Data				
	Is the genotyping procedure described?	Are the primers described?	Were quality control methods used and described?	Are any genotype frequencies previously reported quoted?	Were genotyping personnel blinded to outcome status?	Is extent of missing data summarized?	Where extent is summarized are reasons for missing data given?	Are checks for missingness at random reported?	Is missing genotype data imputed?	Does paper quote number of patients contributing to each analysis?
Vandel et al. (1999)	YES	NO	NO	NO	NO	YES	N/A	N/A	N/A	YES
Hong et al. (2002)	YES	YES	NO	NO	NO	NO	N/A	N/A	N/A	YES
Mosyagin et al. (2004)	YES	YES	YES	YES	NO	NO	N/A	N/A	N/A	YES
Theisen et al. (2004)	YES	YES	NO	YES	NO	NO	N/A	N/A	N/A	YES
Kohlrausch et al. (2008)	YES	NO	NO	NO	NO	NO	N/A	N/A	N/A	YES
Godlewska et al. (2009)	YES	YES	YES	YES	NO	NO	N/A	N/A	N/A	YES
Le Hellard et al. (2009)	YES	YES	NO	NO	NO	YES	YES	NO	NO	YES
Tiwari et al. (2010)	YES	NO	YES	YES	NO	YES	YES	NO	NO	YES
Lencz et al. (2010)	YES	NO	NO	NO	YES	NO	N/A	N/A	N/A	YES
Kohlrausch et al. (2010)	YES	NO	NO	NO	NO	NO	N/A	N/A	N/A	YES
Jassim et al. (2011)	YES	NO	NO	NO	NO	YES	N/A	N/A	N/A	YES
Choong et al. (2013)	YES	NO	NO	NO	NO	NO	N/A	N/A	N/A	YES
Gagliano et al. (2014)	YES	YES	YES	NO	NO	YES	YES	NO	NO	YES
Dong et al. (2015)	YES	YES	NO	NO	NO	NO	N/A	N/A	N/A	YES
Pouget et al. (2015)	YES	NO	YES	NO	NO	YES	YES	NO	N/A	YES
Quteineh et al. (2015)	YES	NO	NO	NO	NO	NO	N/A	N/A	N/A	YES
Saigi et al. (2016)	YES	NO	YES	NO	NO	YES	YES	NO	NO	YES
Nelson et al. (2018)	YES	NO	NO	NO	NO	NO	N/A	N/A	N/A	YES
Menus et al. (2020)	YES	NO	NO	YES	NO	YES	YES	NO	NO	YES
Nicotera et al. (2021)	YES	YES	NO	NO	NO	NO	N/A	N/A	N/A	YES

Table S2. Quality assessment of included mixed population studies (n = 20)

Study	6. Population Stratification			7. Hardy-Weinberg Equilibrium (HWE)		8. Choice and Definition of Outcomes			Score
	If paper does quote number of patients contributing to analyses does this agree to sample size?	Are tests undertaken for cryptic population stratification?	Is cryptic population stratification adjusted for in the analyses?	Was HWE tested?	Where test undertaken, are SNPs deviating from HWE (or their absence) highlighted and excluded from further analysis?	Does the paper clearly define all outcomes investigated?	Is justification provided for the choice of outcomes?	Are results shown for all outcomes mentioned?	
Vandel et al. (1999)	NO	NO	N/A	NO	N/A	YES	YES	YES	8
Hong et al. (2002)	YES	NO	N/A	NO	N/A	YES	YES	YES	11
Mosyagin et al. (2004)	NO	NO	N/A	YES	YES	YES	YES	YES	13
Theisen et al. (2004)	YES	NO	N/A	YES	YES	YES	YES	YES	11
Kohlrausch et al. (2008)	YES	NO	N/A	YES	YES	YES	YES	YES	12
Godlewska et al. (2009)	YES	NO	N/A	YES	YES	YES	YES	YES	13
Le Hellard et al. (2009)	NO	NO	N/A	YES	YES	YES	YES	YES	14
Tiwari et al. (2010)	YES	NO	N/A	YES	YES	YES	YES	YES	17
Lencz et al. (2010)	YES	NO	N/A	YES	YES	YES	YES	YES	12
Kohlrausch et al. (2010)	YES	NO	N/A	YES	YES	YES	YES	YES	11
Jassim et al. (2011)	YES	NO	N/A	YES	YES	YES	YES	YES	12
Choong et al. (2013)	YES	NO	N/A	YES	YES	YES	YES	YES	13
Gagliano et al. (2014)	YES	NO	N/A	YES	YES	YES	YES	YES	18
Dong et al. (2015)	YES	NO	N/A	YES	YES	YES	YES	YES	14
Pouget et al. (2015)	YES	NO	N/A	YES	YES	YES	YES	YES	16
Quteineh et al. (2015)	NO	NO	N/A	YES	YES	YES	YES	YES	11
Saigi et al. (2016)	NO	NO	N/A	YES	YES	YES	YES	YES	14
Nelson et al. (2018)	YES	NO	N/A	YES	YES	YES	YES	YES	11
Menus et al. (2020)	NO	NO	N/A	NO	N/A	YES	YES	YES	11
Nicotera et al. (2021)	YES	NO	N/A	YES	NO	YES	YES	YES	11

NO = Not mentioned in the manuscript or in the Method(s) paper that the authors referenced. N/A = Not Applicable as the answer to the main question is "No".

The assessment tool was adapted from Jorgensen and Williamson (2008).
